# Integrated Lead/Iodine Management for Sustainable Perovskite Solar Modules

**DOI:** 10.1002/adma.202518752

**Published:** 2026-01-05

**Authors:** Guo‐Bin Xiao, Niansheng Xu, Zhen‐Yang Suo, Sibei Mai, Dandan Hu, Feng Gao, Jing Cao

**Affiliations:** ^1^ State Key Laboratory of Natural Product Chemistry Key Laboratory of Nonferrous Metal Chemistry and Resources Utilization of Gansu Province College of Chemistry and Chemical Engineering Lanzhou University Lanzhou P. R. China; ^2^ Department of Physics Chemistry and Biology Linköping University Linköping Sweden; ^3^ Wallenberg Initiative Materials Science for Sustainability Department of Physics Chemistry and Biology Linköping University Linköping Sweden

**Keywords:** environmental safety, lead/iodine management, perovskite, recycling, solar module

## Abstract

The environmental hazards posed by the release of toxic lead ions (Pb^2+^) and volatile iodine species remain a major obstacle to the large‐scale commercialization of perovskite solar modules. Here, we propose a dual‐function adsorbent–porphyrin‐modified whitlockite nanocomposites (WH&Por), which simultaneously captures volatile iodine via surface‐coated porphyrin and adsorbs Pb^2+^ through the whitlockite matrix, forming an integrated pollutant barrier throughout the entire lifecycle of perovskite devices. Experimental results show that even under severe mechanical damage with a WH&Por loading of just 0.25 mg/cm^2^, the protection system prevents the leakage of lead and iodine, demonstrating excellent environmental safety performance. In addition, we developed a semi‐closed loop recycling process that enables the recovery of high‐purity PbI_2_ from perovskite waste, achieving a recovery yield of up to 96.9%. Devices reassembled using the recovered PbI_2_ exhibit power conversion efficiencies comparable to those of their pristine counterparts. Remarkably, the residual Pb^2+^ concentration in the treated recycling waste solution was reduced to below 10 ppb, well beneath the stringent limit set by the European Union Drinking Water Directive 98/83/EC. This study offers a practical and integrated “protection–recycling” solution to one of the key environmental challenges facing perovskite photovoltaics.

## Graphical abstract

Perovskite solar modules face environmental risks from lead and iodine leakage. A dual‐function adsorbent—porphyrin‐modified whitlockite nanocomposites—effectively captures iodine and Pb^2^^+^, even under severe damage. Combined with a semi‐closed recycling process, it recovers 96.9% high‐purity PbI^2^ and reduces residual Pb^2^^+^ to <10 ppb, offering an integrated “protection–recycling” solution for sustainable perovskite photovoltaics.

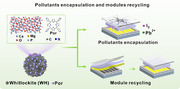

## 1 Introduction

Photovoltaic (PV) technologies, including silicon‐based solar cells [1, 2], dye‐sensitized solar cells (DSSCs) [3, 4], organic photovoltaics (OPVs) [5, 6], cadmium telluride (CdTe) cells [7, 8], copper indium gallium selenide (CIGS) cells [9, 10], and perovskite solar cells (PSCs) [11, 12, 13, 14], have garnered considerable attention for their potential to diversify and reshape the landscape of sustainable energy solutions. Among these, PSCs have emerged as particularly promising candidates, offering outstanding power conversion efficiencies alongside low‐cost and scalable fabrication processes [15, 16, 17, 18]. However, during device operation, the leakage of water‐soluble lead ions from perovskite materials poses significant risks to both environmental ecosystems and human health [19, 20, 21, 22]. Additionally, intrinsic chemical instabilities within the perovskite structure can lead to the breakdown of Pb─I bonds, rendering iodide ions (I^−^) highly reactive and promoting the formation of volatile molecular iodine (I_2_) [23, 24, 25, 26]. The release of I_2_ not only inflicts severe damage on ecosystems but also poses serious health risks to humans [27, 28, 29]. Iodine excess poses substantial risks to thyroid health and can adversely affect the cardiovascular, neurological, and renal systems, while also exerting pronounced impacts on soil ecosystems. This situation is anticipated to be further aggravated by the rapid expansion of PV technologies, resulting in the substantial accumulation of end‐of‐life PV modules. Thus, effective control of toxic Pb ions and volatile iodine species is key to preventing hazardous release, enabling device recycling, and ensuring sustainable PSC development.

Pollutant encapsulation strategies have predominantly targeted toxic lead ions in damaged and end‐of‐life devices [30, 31, 32]. Strategies involving physical encapsulation and chemical adsorption such as cross‐linked supramolecular complexes, macrocyclic porphyrin molecules, and cation‐exchange resins, have been developed to immobilize Pb^2+^ and suppress leakage under harsh environmental conditions [33, 34, 35]. A dual‐sided lead‐sequestration approach, combining a transparent phosphonic acid‐based molecular film on the front glass and a polymer layer embedded with lead‐chelating agents on the rear side, has demonstrated over 96% efficiency in capturing lead released from fractured devices [36]. Recycling approaches are also gaining prominence, offering the dual benefits of mitigating lead‐related environmental risks and minimizing material loss [37, 38, 39, 40, 41]. For instance, iron‐doped hydroxyapatite, with its strongly negative surface charge, enables selective Pb^2+^ recovery through enhanced electrostatic interactions [42]. Moreover, aqueous‐based recycling methods have been developed to regenerate degraded perovskite films while recovering functional layers such as charge transport materials, substrates, cover glasses, and electrodes [43]. Nonetheless, the challenge of volatile iodine species remains largely unresolved. Future efforts should focus on the integrated management of lead ions and volatile iodine to enable simultaneous containment and recovery of hazardous substances and device components, thereby advancing the environmental and material sustainability of perovskite photovoltaics.

**FIGURE 1:**
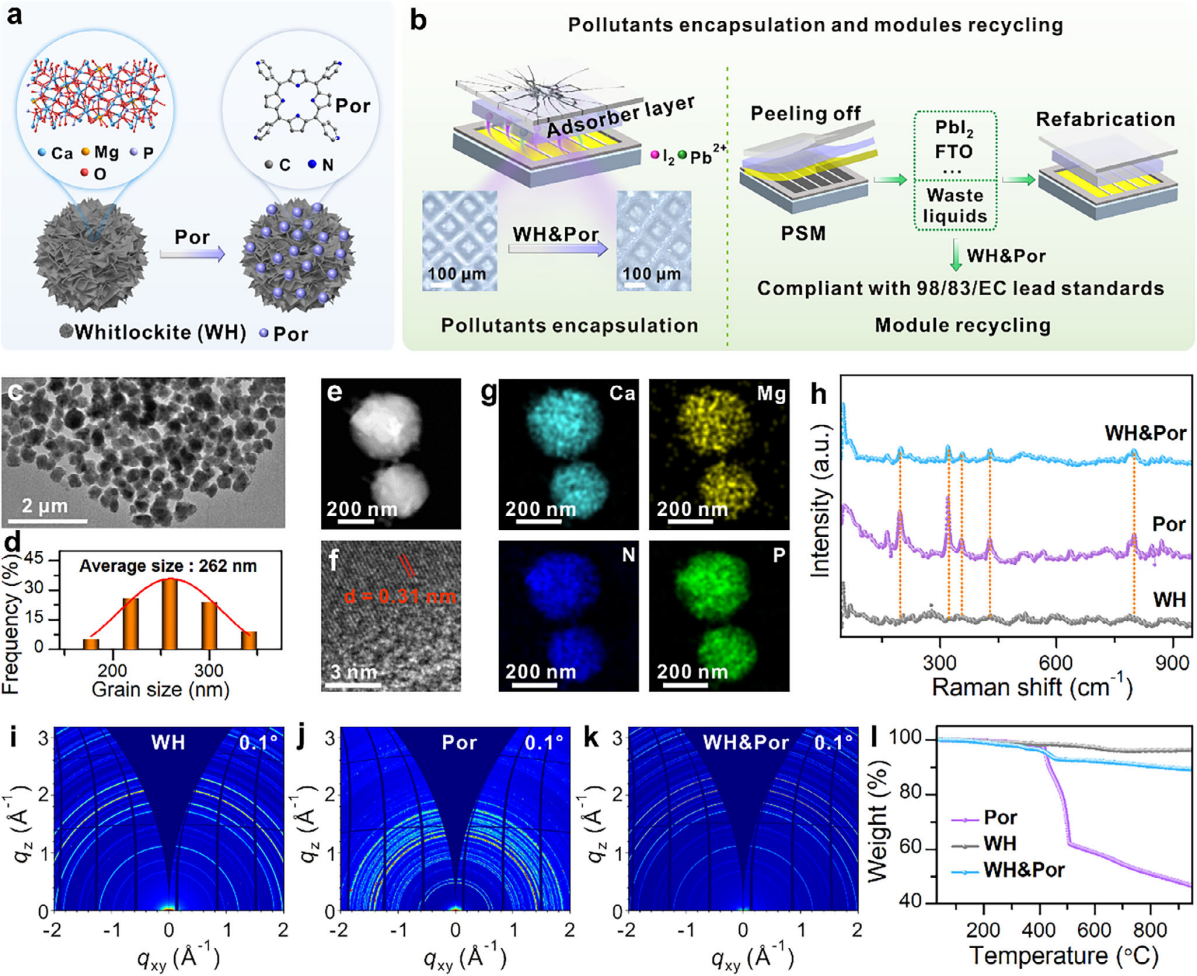
(a) Schematic illustration of WH&Por synthesis. (b) Conceptual diagram of WH&Por for pollutants encapsulation and module recycling. TEM image (c) and particle size distribution (d) of WH&Por. (e, f) High‐resolution TEM image showing lattice fringes of WH&Por. (g) Elemental distribution via EDS mapping. (h) Raman spectra of WH, Por, and WH&Por samples. GIWAXS patterns of WH (i), Por (j) and WH&Por (k). (l) Thermal stability analysis of WH, Por, and WH&Por via TGA.

Here, we propose an integrated lead and iodine management strategy for sustainable perovskite solar modules (PSMs) using porphyrin‐functionalized whitlockite (WH) nanoparticles (Figure 1a). Resulting WH&Por hybrid exhibits dual capture capabilities: surface‐coated porphyrin enables efficient iodine adsorption, while the WH matrix shows high Pb^2+^ affinity. PSMs coated with WH&Por adsorbent layer display no detectable Pb^2+^ or iodine leakage under severe mechanical stress, ensuring environmental safety. A stepwise acidification/alkalization process followed by HI treatment enables 96.9% recovery of PbI_2_ from adsorbent and degraded perovskite layers in end‐of‐life modules. Subsequent WH&Por treatment reduces residual Pb^2+^ in the final waste to <10 ppb, meeting the discharge limits for lead specified in 98/83/EC (Figure 1b). Refabricated devices achieve a PCE of 23.6% (5  ×  5 cm^2^), comparable to pristine modules, underscoring the scalability and environmental viability of this semi‐closed loop strategy.

## 2 Results and Discussion

### 2.1 Synthesis and Characterization of WH&Por Nanoparticles

The WH&Por nanoparticles were synthesized through a two‐step process (Figure 1a; Figure S1). During device encapsulation, the WH&Por layer was subsequently applied on top of the metal electrode (Figure 1b), serving as an external protective barrier that suppresses Pb^2+^ and iodine leakage and enhances the environmental safety of the module. Initially, WH nanoparticles with a dominant (110) plane were prepared through precipitation‐induced crystallization by gradually adding a phosphate solution into a mixed suspension of calcium and magnesium hydroxides (Figures S2 and S3). Subsequently, Por sample was introduced into the WH dispersion to enable surface functionalization, yielding WH&Por nanoparticles. Transmission electron microscopy (TEM) images revealed that WH&Por nanoparticles exhibit uniform morphology with an average particle diameter of ∼262 nm (Figure 1c,d). High‐resolution TEM identified a lattice spacing of 0.31 nm corresponding to the (0 0 12) plane of WH (Figure 1e,f). Energy‐dispersive X‐ray spectroscopy (EDS) mapping confirmed the homogeneous distribution of Por on the surface of WH nanoparticles (Figure 1g). Compared to pristine WH, the Raman spectrum of WH&Por displayed additional peaks at 197, 320, and 428  cm^−1^, corresponding to vibrational modes of the pyrrolic units in the porphyrin core, along with characteristic bands at 355 and 800 cm^−1^ attributed to the pyridyl substituents of Por [44] (Figure 1h). Fourier transform infrared (FT‐IR) spectra further confirmed the presence of porphyrin species on the WH surface, and the characteristic peaks of Por remained essentially unchanged after hybridization (Figure S4), suggesting that the interaction between WH and Por is dominated by physical adsorption rather than chemical bonding. These spectral features collectively confirmed the successful surface functionalization of WH with Por molecules. X‐ray diffraction (XRD, Figure S5) and grazing‐incidence wide‐angle X‐ray scattering (GIWAXS, Figure 1i–k; Figure S6) analyses revealed no distinct diffraction peaks attributable to crystalline Por, suggesting its amorphous adsorption on the surface. Thermogravimetric analysis (TGA, Figure 1l) confirmed that WH&Por nanoparticles have high thermal stability up to 400°C, supporting their potential as robust candidates for hazardous species capture and recovery in perovskite photovoltaics. In addition, thermal cycling tests (25°C–85°C) further verified their excellent structural integrity (Figure S7), as no discernible changes in the XRD patterns were observed after repeated heating‐cooling cycles, confirming the outstanding robustness of WH&Por under temperature fluctuations.

### 2.2 Adsorption Performance Analyses of WH&Por Toward Pb^2+^ and I_2_

**FIGURE 2:**
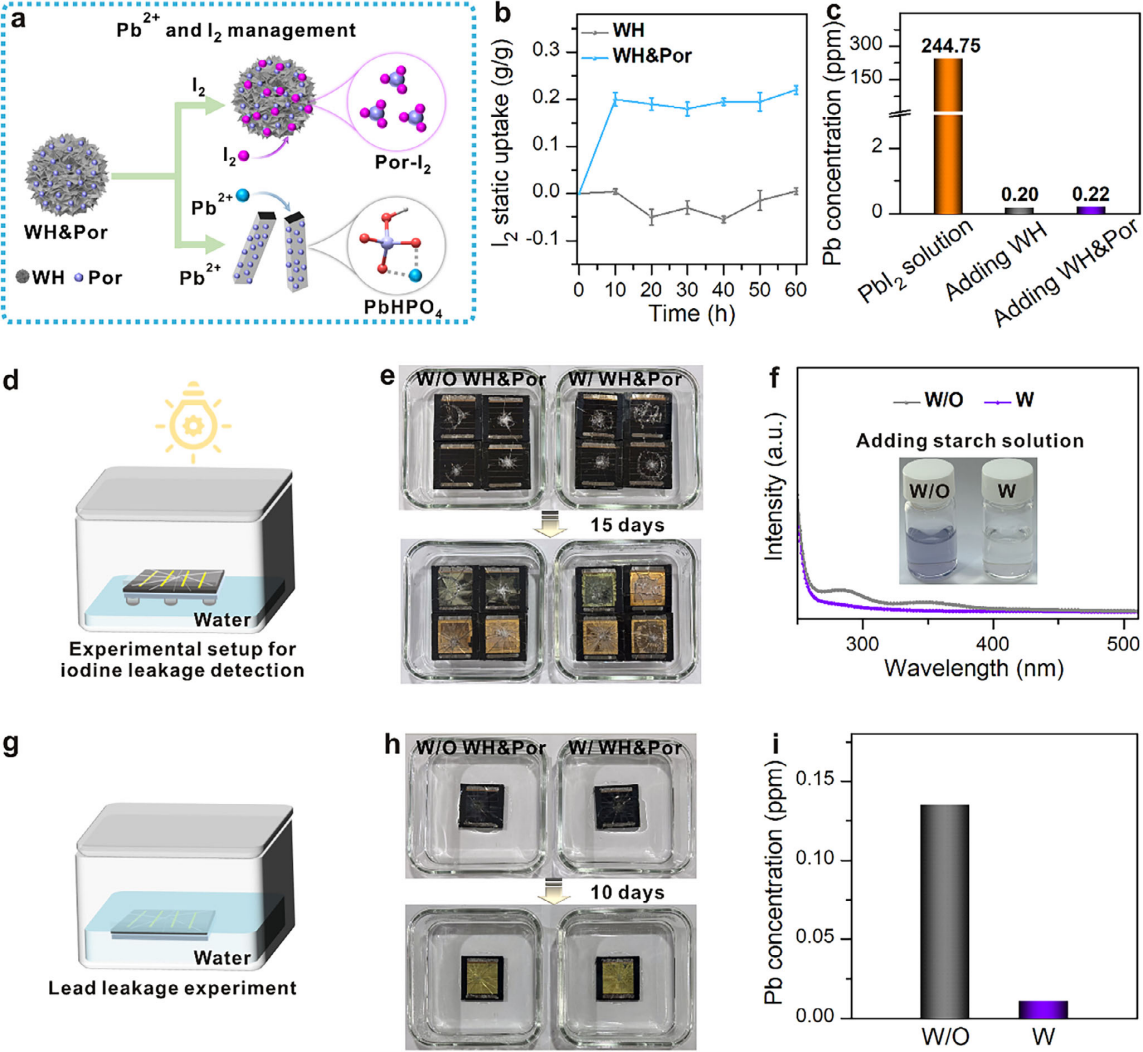
(a) Schematic illustration of the dual‐function adsorption mechanism of WH&Por for I_2_ and Pb^2+^. (b) I_2_ vapor uptake profiles of WH and WH&Por at 348 K. (c) Pb^2+^ adsorption capacity of WH and WH&Por. (d) Experimental setup for I_2_ adsorption evaluation under illumination and water exposure. (e) Images of damaged PSMs before and after 15 days of illumination, along with corresponding images after the addition of starch solution. (g) Schematic of Pb^2+^ leaching test apparatus. (h) Photographs of damaged PSMs immersed in deionized water for various durations. (i) Quantitative analysis of Pb^2+^ concentrations in leachates over time.

The dual functionalities of WH&Por nanoparticles for simultaneous adsorption of volatile iodine and lead ions were first evaluated (Figure 2a). To assess the iodine adsorption capacity, both WH&Por and WH nanoparticles were exposed to I_2_ vapor at 348 K. As shown in Figure 2b, WH&Por reached saturation within ∼10 h, achieving a maximum iodine uptake of 0.20 g g^−1^, whereas WH exhibited negligible adsorption, confirming the essential role of Por in iodine capture (Figure S8). Time‐resolved tests show that WH&Por begins capturing volatilized I_2_ almost immediately (Figure S9), indicating a response fast enough for real‐time leakage suppression. The adsorption mechanism was probed by X‐ray photoelectron spectroscopy (XPS), FT‐IR, UV–vis and semiempirical ab initio molecular dynamics (SE‐AIMD). After I_2_ exposure, XPS shows new I 3d peaks at 629.9/618.2 eV and a +0.1–0.2 eV shift of pyrrolic/pyridyl N 1s, evidencing weak coordination/limited charge transfer between iodine and nitrogen sites (Figure S10a,b) [29, 45]. FT‐IR further supports coordination: the N─H stretch (3310 cm^−1^) diminishes, while porphyrin bands shift (pyridyl C═C 1593→1621 cm^−1^, C─N 969→981 cm^−1^, pyrrolic C─H 800→790 cm^−1^) (Figure S11) [46, 47]. Consistently, UV–vis shows only minor spectral changes on mixing Por with I_2_ (Figure S12), corroborating limited charge transfer. SE‐AIMD reveals a distance‐dependent interaction: long‐range π‐dispersion guides I_2_ toward the macrocycle, transitioning to directional pyridyl–I_2_ stabilization at the binding site (reduced mean‐square displacement (MSD); interaction‐energy profiles in Figure S13a–d). Collectively, WH&Por captures iodine via coordination‐dominated binding with limited charge transfer, delivering rapid and efficient uptake. The adsorption performance of WH&Por toward Pb^2+^ was further evaluated. Specifically, 10 mg of WH&Por nanoparticles were dispersed in 80 mL of an aqueous PbI_2_ solution and left undisturbed for 1 h. After centrifugation, the supernatant was collected for inductively coupled plasma mass spectrometry (ICP‐MS) analysis. As shown in Figure 2c, the Pb^2+^ concentration markedly decreased from 244.75 to 0.22 ppm upon treatment with WH&Por, corresponding to a Pb^2+^ adsorption capacity of 1.96 g g^−1^. This value is comparable to that of pristine WH (Figure 2c), indicating that WH&Por retains the inherent lead‐adsorbing capability of the WH matrix.

For device integration, WH&Por nanoparticles were solution‐coated onto polyolefin elastomer (POE) films. Metallographic microscopy shows that the coating preferentially deposits along the grid regions (Figure S14). We selected a loading of 0.25 mg cm^−2^, which provides sufficient capacity to capture the full Pb^2+^ release from a module while balancing protection and material use. The coated film serving as the adsorbent layer was then laminated onto the metal electrode to complete the encapsulation (Figure S15). To simulate operational stress, devices with and without WH&Por were mechanically damaged and placed in sealed chambers filled with deionized water, followed by continuous illumination (Figure 2d). The mechanical damage was introduced by dropping ice balls onto the mini‐modules to mimic hail impact under real outdoor conditions, following the IEC 61215‐2:2021 standard for photovoltaic panel hail‐impact testing. After 15 days of illumination, both devices with and without the WH&Por encapsulation layer exhibited evident degradation and yellowing (Figure 2e; Figures S16 and S17). Given that I_2_ readily reacts with mobile I^−^ ions in water to form triiodide ions (I_3_^−^) [48]. UV–vis tests were employed to analyze the aqueous solutions collected from the test chambers (Figure 2f). The control devices showed a pronounced absorption peak at 350 nm, characteristic of I_3_^−^, whereas the WH&Por‐encapsulated devices exhibited negligible absorbance at this wavelength, indicating effective inhibition of iodine release. Triiodide titration further quantified the release as ∼1.08 ppm for controls versus <limit of detection for WH&Por (Figure S18). Furthermore, upon addition of starch solution into the aqueous solutions collected from the test chambers, the control sample turned light purple due to the presence of iodine, while the WH&Por‐treated sample remained colorless (inset, Figure 2f), further confirming the high iodine adsorption efficiency of WH&Por under simulated operational conditions. These results underscore that the iodine management strategy in this work focuses on suppressing the release of volatile iodine species during device operation and failure, thereby mitigating environmental contamination and reinforcing the comprehensive environmental protection capability of the encapsulated perovskite system.

The Pb^2+^ leakage from mechanically damaged PSMs was further assessed under simulated operational conditions. As illustrated in Figure 2g, modules (5.0 × 5.0 cm^2^) with and without WH&Por encapsulation were deliberately damaged and immersed in 80 mL of deionized water in sealed glass chambers (Figure 2h). After 10 days of soaking, both sets of devices exhibited visible degradation, including yellow discoloration (Figure 2h). ICP‐MS analysis revealed that the control sample released up to 0.14 ppm of Pb^2+^ into the solution, while the WH&Por‐encapsulated module limited Pb^2+^ leakage to just 0.01 ppm (Figure 2i). To further assess environmental robustness, we conducted leaching tests in mildly acidic water (pH ≈ 4.5) to mimic acid rain conditions (Figure S19). Even under these conditions, the WH&Por encapsulation markedly suppressed the release of both Pb^2+^ and I_2_, maintaining strong protection. These results confirm the strong lead‐immobilizing capability of WH&Por. Combined with its demonstrated iodine adsorption, WH&Por nanoparticles serve as a dual‐functional encapsulation material, effectively suppressing both Pb^2+^ and I_2_ pollutants release, thereby offering a promising strategy for environmentally sustainable devices.

### 2.3 Recycling of Adsorbent and Electrode Components

**FIGURE 3:**
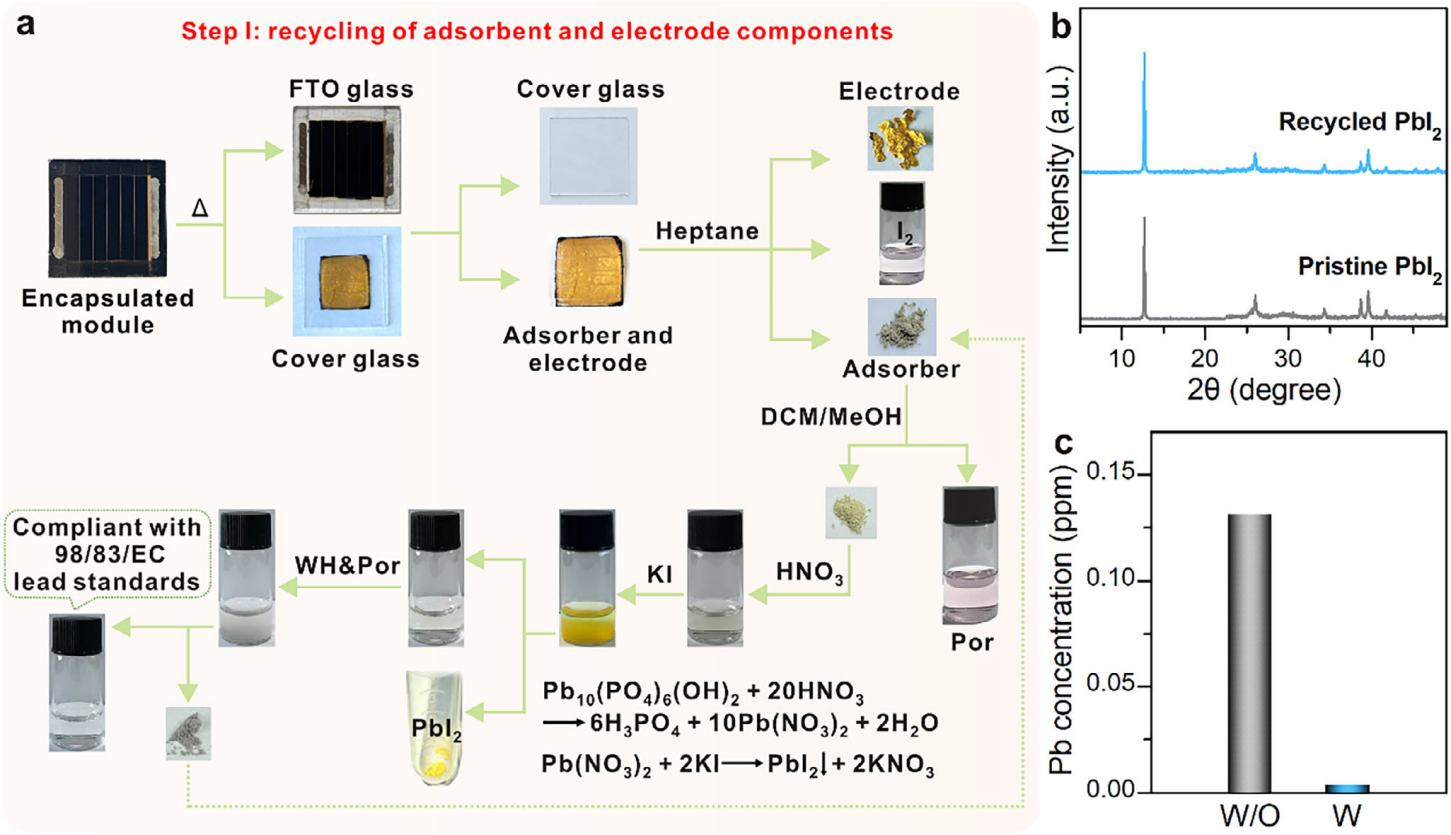
(a) Photographs illustrating thermal delamination of encapsulated PSMs, recovery of metal electrodes, and the process for converting WH&Por‐adsorbed Pb^2+^ into PbI_2_ via precipitation, with corresponding chemical reactions. (b) XRD patterns of pristine and recycled PbI_2_. (c) Pb^2+^ concentrations in residual solution before and after WH&Por treatment.

Efficient recovery of degraded components from end‐of‐life PSMs is critical to advancing the environmental and material sustainability of perovskite photovoltaics. In this study, PSMs were constructed on fluorine‐doped tin oxide (FTO) glass substrates and encapsulated with an elastomeric sealant and a protective cover glass. Previous studies have shown that thermal activation facilitates the disassembly of encapsulated PSMs, allowing for the effective recovery of intact FTO and cover glass components [41]. Accordingly, heating the devices at 200°C for 3 min softened and melted the butyl rubber encapsulation along the module edges, generating interfacial strain that promoted delamination at the interface between the hole‐transport material (HTM) and metal electrode (Figure 3a). As a result, the electron transport material (ETM) and perovskite layers remained adhered to the FTO substrate, while the HTM, metal electrode, and adsorbent layer on the protective cover glass were mechanically separated. The detached layers on the cover glass side were subsequently rinsed with dichloromethane to recover a clean cover glass and the adsorbent layer containing the metal electrode. The recovered adsorbent and electrode were then subjected to ultrasonic treatment in heptane, followed by centrifugation, enabling the separation and recovery of metal electrode and WH&Por nanoparticles.

The WH&Por‐based adsorbent was subjected to a multi‐step recovery process. Initially, the spent adsorbent was washed with heptane and centrifuged to separate the solid component. The dissolved iodine was extracted into the heptane phase, as evidenced by a distinct absorption peak at 294 and 350 nm (Figure S20), characteristic of triiodide (I_3_^−^) [24, 48]. The remaining solid was subsequently washed with CH_2_Cl_2_/CH_3_OH (DCM/MeOH, v/v = 3/1), after which the Por was recovered by DCM/MeOH extraction (Figure S21). The solid residue was analyzed to determine the structural transformation post‐Pb^2+^ adsorption. XRD patterns revealed the formation of hydroxypyromorphite [49], a thermodynamically stable lead phosphate mineral (Figure S22). TEM imaging and EDS mapping further confirmed the presence of nanowire‐shaped hydroxypyromorphite (Figure S23). The Pb^2+^ adsorption by WH proceeds via an acid‐assisted dissolution–recrystallization pathway: slight proton‐catalyzed dissolution of WH releases phosphate, which then reacts with Pb^2+^ to precipitate hydroxypyromorphite on/near the WH surface (Figure S24) [49]. To recycle the adsorbed Pb^2+^, the lead‐laden WH&Por solid was dissolved in nitric acid to release Pb^2+^ ions into solution. Upon the addition of potassium iodide, a yellow precipitate of PbI_2_ formed instantly (Figure 3a). The PbI_2_ was collected via centrifugation, washed with ethanol, and dried. To eliminate any residual Pb^2+^ in the supernatant, an additional adsorption step using fresh WH&Por was conducted. Post‐adsorption, the solution underwent centrifugation, and the Pb^2+^‐loaded WH&Por was redirected into the PbI_2_ recovery cycle, enabling a semi‐closed loop recycling system. XRD analysis confirmed that the regenerated PbI_2_ retained the same diffraction pattern as pristine PbI_2_, indicating preserved crystallinity (Figure 3b). ICP analysis of the solution before and after WH&Por treatment showed a significant drop in Pb^2+^ concentration from 0.131 to 0.003 ppm, well below environmental safety limits (Figure 3c). Collectively, these results demonstrate that WH&Por effectively prevents Pb^2+^ leakage from PSMs and enables efficient semi‐closed loop recovery of toxic lead species into reusable PbI_2_ precursors for device re‐fabrication.

### 2.4 Recovery of Degraded Perovskite Layer and TCO from End‐of‐Life PSMs

**FIGURE 4:**
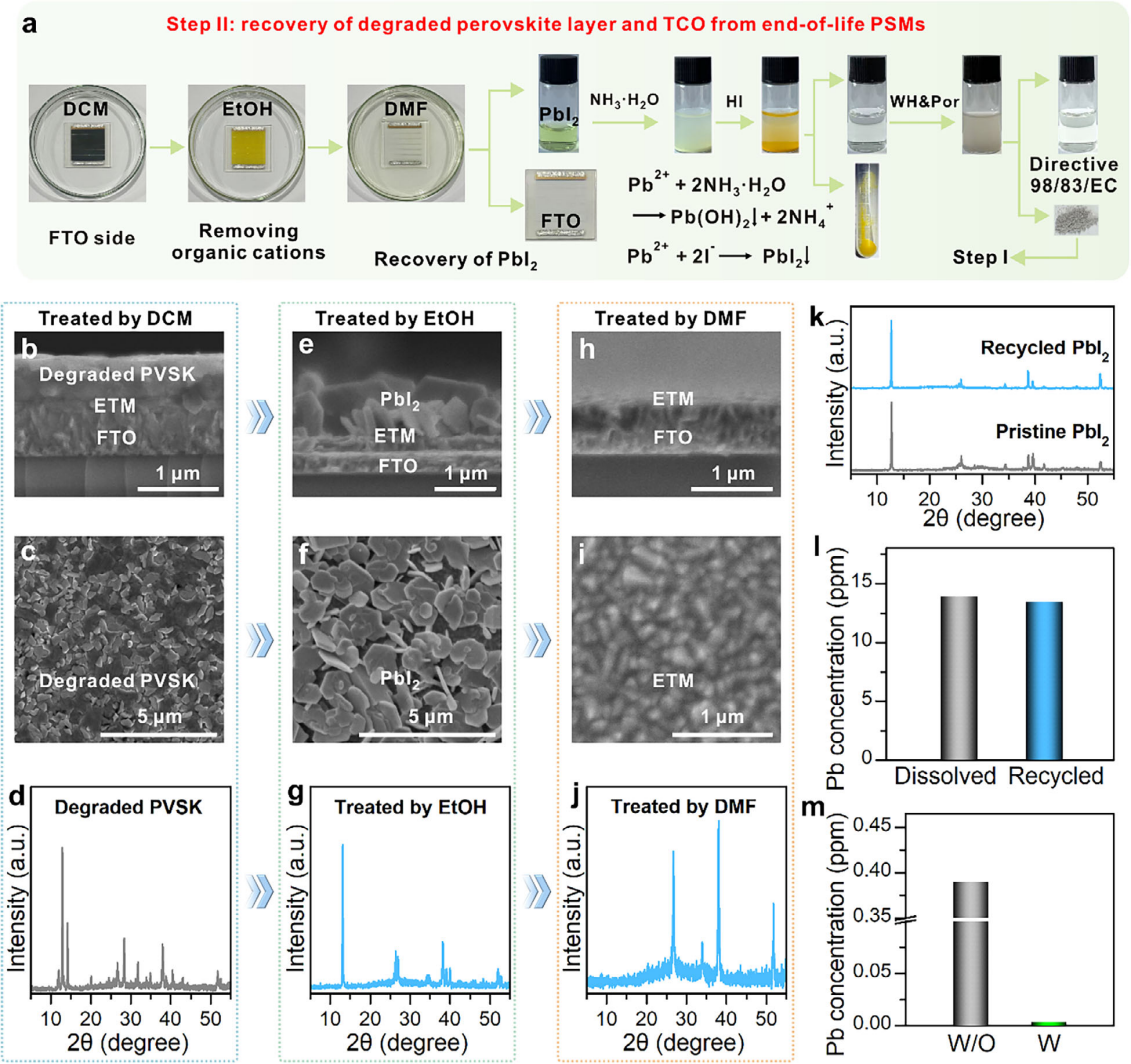
(a) Schematic diagram illustrating the recovery of substrates, PbI_2_, along with the corresponding reactions. Cross‐sectional (b), surface (c) SEM images, and XRD (d) of end‐of‐life perovskite (PVSK) film after metal electrode and HTM removal. Cross‐sectional (e), surface (f) SEM images, and XRD (g) of perovskite film post‐ethanol soaking. Cross‐sectional (h), surface (i) SEM images, and XRD (j) of the residual substrate after PbI_2_ dissolution in DMF. (k) XRD patterns comparing pristine and recycled PbI_2_. (l) Pb^2+^ concentration in dissolved PSMs and recovered PbI_2_ in equal amounts of DMF. (m) Pb^2+^ concentration in wastewater before and after treatment with WH&Por following PbI_2_ recovery.

The FTO side adhered ETM and perovskite layers were further used to stepwise recover all high‐value components (Figure 4a). SEM cross‐sectional and surface imaging reveal that the perovskite film was destroyed with pronounced holes and extensive flake‐like structures (Figure 4b,c). XRD analysis further confirms significant degradation of the perovskite film, with the emergence of PbI_2_ products (Figure 4d). The substrate was immersed in ethanol to promote the decomposition of the perovskite and the dissolution of organic cations. Cross‐sectional and surface SEM images reveal that the perovskite film undergoes complete decomposition upon exposure to ethanol, transforming into a flake‐like morphology (Figure 4e,f), with the organic components dissolved into the solution (Figure S25). XRD confirms the complete conversion of the perovskite phase into PbI_2_ (Figure 4g). The obtained substrate was further treated with N,N‐dimethylformamide (DMF) to enable the dissolution of PbI_2_, allowing simultaneous recovery of PbI_2_ and a clean FTO substrate with preserved ETM layers (Figure 4h,i). XRD further confirms the removal of PbI_2_ from the substrate (Figure 4j).

To recover PbI_2_ from the DMF extract, a selective precipitation approach was adopted. Upon the addition of aqueous ammonia (NH_3_·H_2_O), Pb^2+^ initially formed a complex, which was subsequently reprecipitated upon the introduction of iodide ions (Figure 4a) [41, 50]. The resulting yellow precipitate was collected by centrifugation, washed with ethanol, and dried to afford high‐purity PbI_2_. Following PbI_2_ recovery, the remaining lead‐containing waste solution was treated with WH&Por to selectively adsorb residual Pb^2+^. The Pb^2+^‐adsorbed WH&Por was then separated from the solution and subjected to the same regeneration and PbI_2_ recovery protocol described in Step I (Figure 3a), thereby integrating the material into a semi‐closed loop recycling process. XRD and UV–vis spectroscopy analyses confirmed that the recovered PbI_2_ possessed identical crystallographic and optical characteristics compared to commercial PbI_2_ (Figure 4k; Figure S26). Both the TGA and FT‐IR analyses show results nearly identical to those of pristine PbI_2_ (Figures S27 and S28), confirming the absence of residual organic species in the recovered material. ICP analysis determined a PbI_2_ recovery yield of 96.9% (Figure 4l). Furthermore, WH&Por effectively reduced the residual Pb^2+^ concentration in the waste stream from 0.39 ppm to 4 ppb, significantly below regulatory discharge thresholds (10 ppb) (Figure 4m).

The recycling of FTO substrates is critical for reducing the overall material cost of PSMs, as FTO constitutes a significant portion of the costs [51, 52]. The SnO_2_‐coated FTO substrates were recycled by sequential cleaning and UV‐ozone treatment to eliminate surface defects in the SnO_2_ layer (Figure S29) [43, 53]. GIWAXS was employed to analyze the crystallographic structure of both pristine and recycled FTO/ETM substrates. The diffraction patterns observed in Figure S30 were nearly identical for both pristine and recycled substrates, which was further corroborated by the XRD tests (Figure S31). Conductive atomic force microscopy (C‐AFM) measurements indicated comparable current profiles for recycled (8.0 nA) and pristine (7.8 nA) FTO/ETM substrates, confirming that the charge transport performance was preserved (Figure S32). Transmittance tests revealed minimal differences between recycled and pristine FTO/ETM substrates, supporting the complete removal of the residues (Figure S33). These results demonstrate that all key components of PSMs including the electrodes, PbI_2_, and FTO/ETM substrates, can be efficiently recovered using a solution‐processable, environmentally responsible protocol. This method lays the foundation for the sustainable, semi‐closed loop manufacturing of perovskite photovoltaics.

### 2.5 Refabrication of PSMs Using Recycled Key Components

**FIGURE 5:**
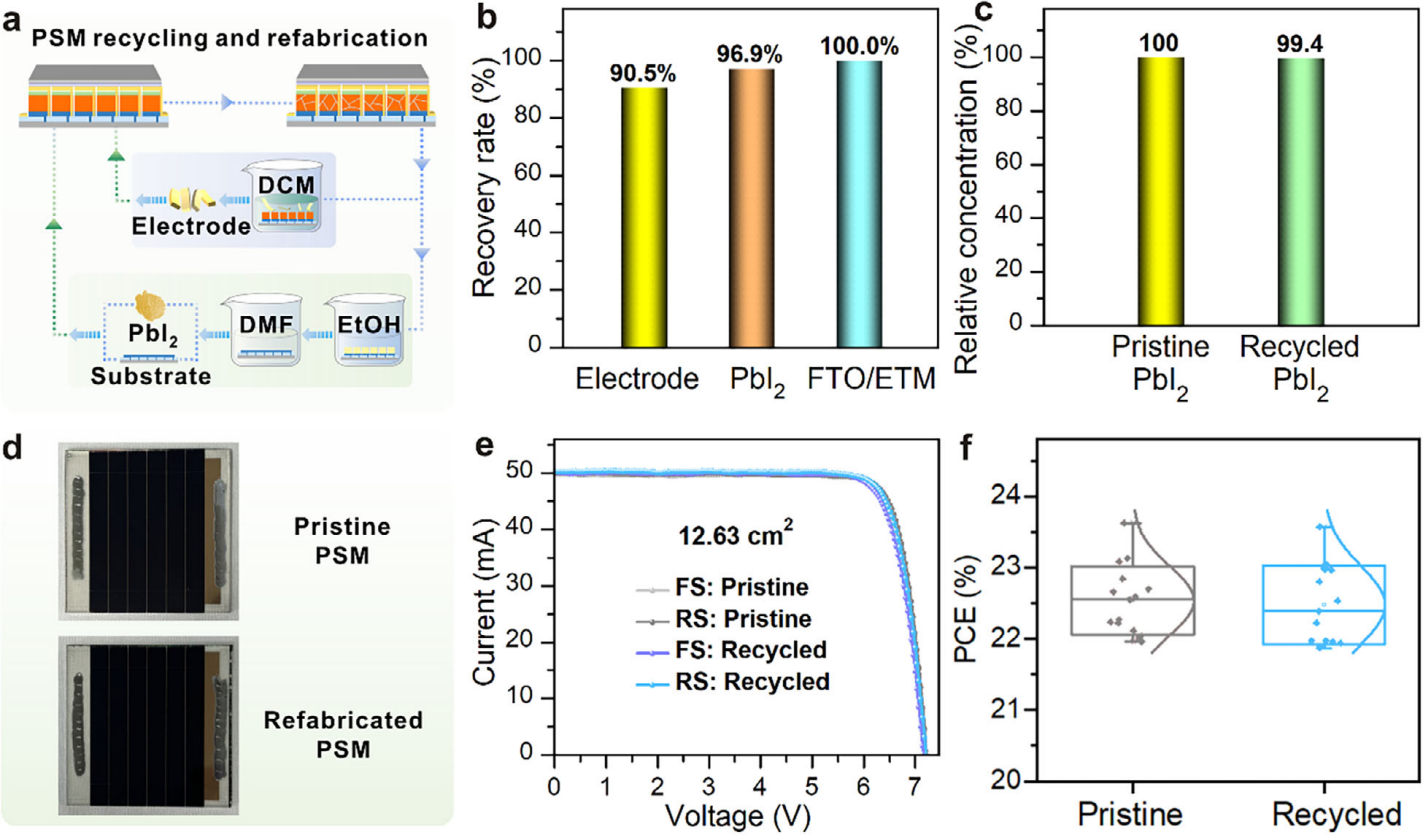
(a) Schematic diagram illustrating the recycling and refabrication of PSMs. (b) Recovery rates of key materials in PSMs. (c) Relative lead concentration in DMF solution with equal amounts of PbI_2_ from different sources. (d) Photographs of the PSMs with pristine and recycled materials. (e) *I–V* curves of PSMs made with pristine and recycled materials. (f) Statistical efficiency comparison of PSMs with pristine and recycled materials.

The recycling and refabrication of end‐of‐life PSMs offer a promising strategy to reduce production costs and mitigate environmental impact, advancing the sustainable development of photovoltaic technology (Figure 5a). To assess the effectiveness of the proposed recycling protocol, a comprehensive quantitative analysis was conducted on the recovery rates of key functional materials. The recovery rates were found to be 90.5% for the electrode, 96.9% for PbI_2_, and 100% for FTO (Figure 5b). Based on laboratory‐scale fabrication and literature‐reported data [40, 54, 55], the estimated material cost for producing a 5 × 5 cm^2^ perovskite module is approximately 0.854$ (Table S1), while the total cost of the recycling process, including reagents and energy, is about 0.05$ per module (Tables S2 and S3). The recovered FTO/ETM, back glass, and electrode components have an equivalent replacement value of approximately 0.571$, leading to a net material saving of 0.521$ per module, corresponding to a ∼61% reduction in fabrication cost. These findings highlight the strong economic and environmental advantages of the recycling strategy and demonstrate its promising potential for sustainable and scalable perovskite photovoltaic technologies. The purity of the recycled PbI_2_ was evaluated against commercially available high‐purity PbI_2_ (TCI). As shown in Figure 5c, the recycled PbI_2_ retained 99.4% of the relative lead concentration, indicating its exceptional purity and suitability for high‐performance photovoltaic applications. Perovskite films were fabricated using the recycled materials and substrates. XRD and UV–vis absorption spectra revealed that the perovskite films produced from recycled materials exhibited similar crystallinity and optical properties to those prepared from pristine materials (Figures S34 and S35). Additionally, C‐AFM measurements demonstrated that the films fabricated with recycled materials exhibited comparable electrical performance to those made with fresh materials (Figure S36).

PSMs (5 × 5 cm^2^) fabricated using recycled materials demonstrated photovoltaic performance comparable to those made with fresh commercial raw materials (Figure 5d,e; Figure S37 and Table S4). The champion PSMs based on recycled materials achieved a *V_OC_* of 7.21 V, *Isc* of 50.27 mA, *FF* of 82.09%, and a PCE of 23.57% (aperture area of 12.63 cm^2^). In comparison, the device made with fresh materials had a *V_OC_* of 7.22 V, *Isc* of 50.00 mA, *FF* of 82.66%, and a PCE of 23.62%. Steady‐state output tests at the maximum power point under ambient conditions revealed similar operational stability for both sets of devices (Figure S38). Statistical analysis showed that devices using recycled and fresh materials exhibited consistent and repeatable performance across all parameters (Figure 5f; Figure S39). We further evaluated the stability of encapsulated devices fabricated from both pristine and recycled materials (Figure S40). Under accelerated aging conditions of 85°C and 85% RH, both types of devices exhibited excellent operational stability, further confirming the practical value and reliability of the proposed recycling strategy. These findings underscore the feasibility and effectiveness of recycling key materials in PSMs, presenting a promising route to enhance the economic and environmental sustainability of perovskite solar technology.

## 3 Conclusion

In conclusion, we have demonstrated a comprehensive and effective approach to addressing the environmental challenges associated with perovskite photovoltaics by incorporating porphyrin‐coated whitlockite (WH&Por) nanoparticles as dual‐function scavengers for lead and iodine. The surface‐bound porphyrin efficiently captures molecular iodine (0.2 g g^−1^), while the whitlockite matrix effectively adsorbs Pb^2+^ (1.96 g g^−1^), mitigating the release of hazardous species during mechanical damage. The integration of just 0.25 mg/cm^2^ of WH&Por nanoparticles as an adsorbent layer in modules (12.63 cm^2^) significantly reduces Pb and iodine release. Moreover, a stepwise recovery protocol allows for the efficient reclamation of valuable materials, with a 96.9% recovery of PbI_2_ and minimal lead content in the waste solution (<10 ppb), meeting the directive 98/83/EC of the European Union standards. Devices refabricated using the recovered components exhibit photovoltaic performance comparable to pristine modules, highlighting the feasibility and effectiveness of this strategy for enhancing the environmental and economic sustainability of perovskite solar technologies. This approach offers a promising pathway for the safe recycling and long‐term viability of perovskite photovoltaic in commercial applications.

## 4 Experimental Section

### 4.1 Materials

All chemicals were obtained from commercial suppliers and used as received without further purification.

### 4.2 Synthesis of WH&Por

WH was synthesized following a previously reported method. Briefly, 23 mm Mg(OH)_2_ and 77 mm Ca(OH)_2_ were added to 50 mL of deionized water preheated to 80°C under continuous stirring. After 10 min, 50 mL of H_3_PO_4_ (85%) was added dropwise at 12.5 mL/min. The mixture was stirred at 80°C for 24 h to allow aging and crystallization. The resulting precipitate was collected by vacuum filtration, washed with deionized water, and freeze‐dried. To prepare the WH&Por composite, 1 g of WH powder was dispersed in 20 mL of porphyrin solution (5 mg/mL in chloroform/methanol, v/v = 4/1) and ultrasonicated for 3 h. The product was recovered by centrifugation and dried under vacuum.

### 4.3 Device Fabrication

FTO glass substrates (5 × 5 cm^2^) were patterned using a 1064 nm laser scribing system (20 W, 500 mm/s, 400 kHz, 30 ns) to form P1 lines, followed by sequential ultrasonication in acetone, deionized water, and 2‐propanol (10 min each). The SnO_2_ electron transport layer was prepared via a chemical bath deposition method. The perovskite precursor solution (Cs_0.08_FA_0.92_PbI_3_) was prepared in DMF/DMSO (v/v = 4/1) and spin‐coated in a two‐step process (1000 rpm 10 s, then 5000 rpm 30 s), with 200 µL ethyl acetate dynamically dripped during the second step. Films were annealed at 120°C for 40 min and passivated with 20 mM PEAI in IPA. For the HTM, a conventional Spiro‐OMeTAD solution was spin‐coated at 3000 rpm. P2 scribing was performed prior to thermal evaporation of 80 nm gold, followed by P3 scribing and laser edge cleaning. Final modules with six series‐connected sub‐cells had an active area of 12.63 cm^2^. To fabricate encapsulant films, WH&Por composite was dispersed in IPA (5 mg/mL) and blade‐coated. The films were dried under ambient conditions to form uniform encapsulation layers.

## Conflicts of Interest

The authors declare no conflicts of interest.

## Supporting information




**Supporting file**: adma72050‐sup‐0001‐SuppMat.pdf

## Data Availability

The data that support the findings of this study are available from the corresponding author upon reasonable request.;
