# Supplementary material for: Integrated Lead/Iodine Management for Sustainable Perovskite Solar Modules
Source: Adv Mater. 2026 Jan 5;38(11):e18752. doi: 10.1002/adma.202518752 (PMC12921351; doi:10.1002/adma.202518752)
Supplement: Supplementary file 1 — Supporting file: adma72050‐sup‐0001‐SuppMat.pdf [file ADMA-38-e18752-s001.pdf]

Supporting information for

Integrated Lead/Iodine Management for Sustainable Perovskite Solar Modules

Guo-Bin Xiao[1,†], Niansheng Xu[2,†], Zhen-Yang Suo[1], Sibei Mai[1], Dandan Hu[1], Feng Gao[2, 3,*], Jing Cao[1,*]

[1] State Key Laboratory of Natural Product Chemistry, Key Laboratory of Nonferrous Metal Chemistry and Resources Utilization of Gansu Province, College of Chemistry and Chemical Engineering, Lanzhou University, Lanzhou 730000, P.R. China

[2] Department of Physics, Chemistry and Biology, Linköping University, Linköping, Sweden.

[3] Wallenberg Initiative Materials Science for Sustainability, Department of Physics, Chemistry and Biology, Linköping University, Linköping, Sweden.

[†] G.-B. Xiao and N. Xu contributed equally to this work.

E-mail: feng.gao@liu.se; caoj@lzu.edu.cn

Contents

1. **Experimental Section**

2. **Supporting Figures**

3. **Supporting Tables**

4. **References**

1. Experimental Section

1.1 Materials. All chemicals were purchased from chemical companies and used as received without further purification. lead iodine (PbI_2, TCI), calcium hydroxide ($Ca(OH)_2$, Aladdin), magnesium hydroxide ($Mg(OH)_2$, Aladdin), Urea (99.999%, aladdin), tin(II) chloride dihydrate ($SnCl_2·2H_2O$, 98%, Alfa Aesar), thioglycolic acid (TGA, 99%, Sigma-Aldrich), hydrochloric acid (HCl, 37 wt% in H_2O), formamidinium iodide (FAI), methylammonium chloride (MACl), 2,2',7,7'-Tetrakis[N,N-di(4-methoxyphenyl)amino]-9,9'-spirobifluorene (Spiro-OMeTAD), cesium iodide (CsI), phenethylammonium iodide (PEAI) were purchased from Xi'an Yuri Solar Co., Ltd, bis(trifluoromethane)sulfonimide lithium salt (Li-TFSI, Sigma-Aldrich), FK209 Co(III) TFSI salt (Sigma-Aldrich), N,N-dimethylformamide (DMF, anhydrous, 99.8%, Sigma-Aldrich), dimethyl sulfoxide (DMSO, anhydrous, 99.9%, Sigma-Aldrich), 5,10,15,20-tetra(4-pyridyl)porphyrin (Por, J&K), isopropyl alcohol (IPA, anhydrous, Sigma-Aldrich), acetonitrile (ACN, anhydrous, Sigma-Aldrich), chlorobenzene (anhydrous, Sigma-Aldrich), 4-tert-butylpyridine (tBP, Sigma-Aldrich), ethyl acetate (EA, anhydrous, Sigma-Aldrich).

1.2 Synthetic route of whitlockite (WH). WH was synthesized according to previously reported methods[1]. 50 mL of deionized water was heated to 80 °C in a controlled environment. Subsequently, 23 mM of $Mg(OH)_2$ and 77 mM of $Ca(OH)_2$ were added to the heated water (50 mL) under continuous stirring to ensure complete dispersion. The temperature was maintained at 80 °C, and the solution was stirred for an additional 10 minutes. Following this, 50 mL of phosphoric acid (H_3PO_4, 85%) was introduced into the reaction mixture at a constant rate of 12.5 mL/min under vigorous stirring. The reaction was allowed to proceed for 24 hours under continuous stirring at 80 °C to facilitate aging and crystallization. After the reaction, the resulting precipitate was collected by vacuum filtration. The solid product was washed three times with distilled water and then dried by freeze drying.

1.3 Synthetic route of WH&Por nanoparticles. A solution of porphyrin was prepared by dissolving in a mixed solvent of chloroform and methanol (v/v = 4/1) to achieve a

concentration of 5 mg/mL. Subsequently, 20 mL of the porphyrin solution was combined with 1 g of the synthesized WH powder. The mixture was subjected to ultrasonic treatment for 3 hours to ensure thorough dispersion and promote the reaction between the porphyrin molecules and the WH surface. Following the ultrasonic treatment, the reaction mixture was centrifuged to isolate the solid product. Finally, the product was dried under vacuum to yield the porphyrin-functionalized WH composite.

1.4 Preparation of Encapsulation Films. WH&Por powders were dispersed in isopropanol at a concentration of 5 mg mL^{-1} and ultrasonicated for 30 minutes to ensure a homogeneous suspension. The resulting precursor dispersion was then blade-coated onto the surface of a polyolefin elastomer film. After coating, the films were allowed to dry naturally under ambient conditions to obtain the encapsulation layer. For a 5 × 5 cm^2 module, the perovskite film contains approximately 3 mg of active material, while the applied WH&Por encapsulation layer introduces about 3.18 mg of adsorbent. Based on comparative tests using different loading amounts, 0.25 mg·cm^{-2} was determined as the optimized concentration, which provides sufficient adsorption capacity to capture all released Pb^{2+} even in a complete decomposition scenario. Therefore, this loading was chosen as a rational and efficient balance between protection effectiveness and material utilization.

1.5 Device Fabrications. The fluorine-doped tin oxide (FTO) glass substrates (5 × 5 cm^2) were patterned using a 1064 nm laser scribing system (20 W) at 500 mm/s with 400 kHz frequency and 30 ns pulse width to create P1 lines, followed by a rigorous cleaning procedure involving sequential ultrasonication in acetone, deionized water, and 2-propanol for 10 minutes each. The SnO_2 electron transport layer was prepared via a chemical bath deposition method. 275 mg of $SnCl_2·2H_2O$, 1.25 g of urea, 1.25 mL of HCl, and 25 μL of TGA were dissolved in 100 mL of deionized water. FTO substrates were immersed in the solution within a glass vessel and maintained at 90 °C for 6 hours. After the reaction, the FTO/SnO_2 substrates were removed, rinsed with deionized water and IPA for 2 minutes each, and subsequently annealed at 190 °C for 60 minutes. The perovskite precursor solution ($Cs_{0.08}FA_{0.92}PbI_3$) was formulated by dissolving

stoichiometric amounts of CsI (31.18 mg), FAI (237.32 mg), PbI_2 (726.1 mg) and MACl (20.26 mg) in a mixed solvent system comprising 800 μL of DMF and 200 μL of DMSO. The perovskite layer was deposited via a two-step spin-coating process: initially at 1000 rpm (200 rpm/s acceleration) for 10 seconds, followed by 5000 rpm (2000 rpm/s acceleration) for 30 seconds. During the spin-coating process, 200 μL of EA was dynamically dripped onto the substrate to facilitate crystallization. The films were subsequently annealed at 120 °C for 40 minutes to achieve optimal crystallinity. The perovskite surface was passivated by spin-coating a 20 mM PEAI solution in IPA at 5000 rpm. For the hole transport layer (HTL), a conventional Spiro-OMeTAD-based solution consisting of 73 mg Spiro-OMeTAD in 1 mL chlorobenzene, supplemented with 30 μL of tBP, 18 μL of Li-TFSI solution (520 mg/mL in acetonitrile), and 29 μL of FK209 Co(III) TFSI solution (300 mg/mL in acetonitrile), spin-coated at 3000 rpm for 30 seconds. Considering the instability of Spiro-OMeTAD over extended periods, phthalocyanine—a material with significantly enhanced chemical and thermal robustness—was employed as the hole-transport layer for devices subjected to long-term stability evaluation[2]. The phthalocyanine layer was deposited by spin-coating a 15 mg mL^{-1} solution in $CHCl_3$ onto the perovskite film at 4000 rpm for 25 s[2]. P2 lines were scribed prior to gold deposition using 27% laser power (speed, 500 mm/s; frequency, 100 kHz; pulse duration, 30 ns). The gold electrode (80 nm) was thermally evaporated. Following electrode deposition, P3 lines were created using identical scribing parameters. Laser edge cleaning was subsequently performed to ensure electrical isolation. The final modules, comprising 6 interconnected sub-cells, exhibited an active area of 12.63 cm^2.

For the preparation of encapsulant films, the composite material was first dispersed in IPA to form a uniform suspension with a concentration of 5 mg/mL. The suspension was then deposited onto a substrate using a blade-coated technique at a controlled speed of 20 mm/s to ensure uniform thickness and coverage. After coating, the film was allowed to dry naturally under ambient conditions, facilitating the evaporation of the solvent and the formation of a stable film. Encapsulation was

performed by first cutting the encapsulation film and butyl rubber into appropriate sizes. The nitrile rubber was placed around the perimeter of the module device, while the central area was filled with a double layer of encapsulation film. A glass backplate was then placed on top, and the entire structure was laminated using a thermal laminator. The encapsulation process was completed through three pressing cycles under heating at 110 °C.

1.6 Characterization. Current density-voltage (J-V) characteristics were measured under standard AM 1.5 G illumination (100 mW/cm^2) using a solar simulator (SAN-EI) calibrated with a certified silicon reference cell, with current-voltage sweeps controlled by a Keithley 2601B source meter. The morphological characteristics were investigated using complementary microscopic techniques. High-resolution surface imaging was performed using a field-emission scanning electron microscope (FE-SEM, Hitachi SU-4800). Surface topography, roughness and surface current analysis were conducted using an atomic force microscope (Oxford Cypher ES). Optical properties were characterized through UV-vis absorption spectroscopy using a Cary 7000 spectrophotometer. Structural characterization was performed using X-ray diffraction (XRD) analysis on a Rigaku RINT-2500 diffractometer operating with Cu Kα radiation.

1.7 Recycling of perovskite solar modules (PSMs). To delaminate the encapsulated PSMs, the devices were placed on a hot plate at 200 °C for 3 minutes to soften and melt the encapsulant. Once softened, a knife blade was gently inserted at one corner of the module between the FTO substrate and the back cover glass to separate the two glass layers. The electron-transport material (ETM) and perovskite layer remained on the FTO side, while the hole-transport material (HTM) and metal electrode layers detached. The HTM and metal electrodes attached to the perovskite film were recovered by washing with dichloromethane. To recover PbI_2 from the perovskite films, the delaminated FTO substrates were first immersed in ethanol to fully decompose the perovskite layer and remove organic cations. After drying, the substrates were subsequently soaked in DMF to dissolve the PbI_2, yielding a PbI_2-containing DMF solution and a FTO conductive substrate. High-purity PbI_2 was then recovered from the

DMF solution using a previously reported method[3-5]. Upon the addition of aqueous ammonia ($NH_3 \cdot H_2O$), Pb^{2+} initially formed a complex, which was subsequently reprecipitated upon the introduction of iodide ions. The resulting yellow precipitate was collected by centrifugation, washed with ethanol, and dried to afford high-purity PbI_2. To treat the waste solution generated during the PbI_2 recovery process, the WH&Por material was employed to adsorb residual Pb^{2+} ions, ensuring the treated effluent met environmental discharge standards. The SnO_2-coated FTO substrates were recycled by sequential cleaning and ultraviolet-ozone treatment to eliminate surface defects in the SnO_2 layer.

1.8 Methods of theoretical calculation. Semiempirical ab initio molecular dynamics (SE-AIMD) was performed using the GFN2-xTB Hamiltonian[6-7] as implemented in CP2K[8], which provides an efficient and transferable description of dispersion, electrostatics, polarization, and anisotropic halogen interactions in large supramolecular assemblies. The pyridyl-functionalized porphyrin host and the I_2 guest were fully optimized prior to dynamics to ensure well-relaxed initial structures. All AIMD simulations were conducted in the canonical (NVT) ensemble at 300 K using a CSVR thermostat[9] with a relaxation time constant of 50 fs. A time step of 0.2 fs was employed, and each trajectory was propagated for a total simulation time of 20 ns, allowing sufficient sampling of both the pre-association and bound-state regimes. Long-range dispersion interactions were accounted for through the built-in D4 correction[10], intrinsic to the GFN2-xTB parameterization and validated extensively for halogen bonding, π-stacking, and other noncovalent interactions[6, 10]. At every MD step, total electronic energies and host–guest interaction components were evaluated using CP2K's energy-decomposition workflow[8], enabling direct separation of the pyridyl–I_2 and macrocycle π–I_2 contributions. A periodic simulation cell with at least 15 Å of vacuum padding was applied in all directions to eliminate spurious image interactions. All simulations employed the reference GFN2-xTB parameters distributed with CP2K. This computational protocol follows established semiempirical AIMD methodologies used for supramolecular recognition, ion transport, and halogen-bonded systems[6-7, 10],

providing reliable dynamical sampling at a fraction of the computational cost of full DFT-based AIMD.

2. Supporting Figures

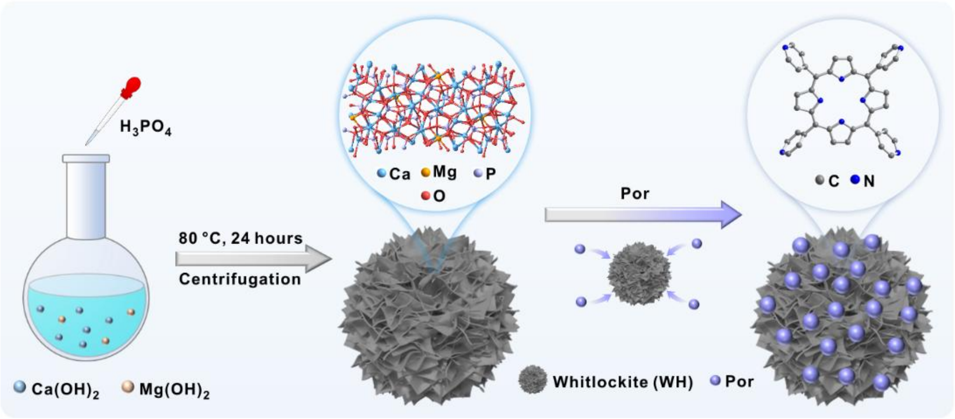

Figure S1. Schematic of the preparation steps of WH&Por.

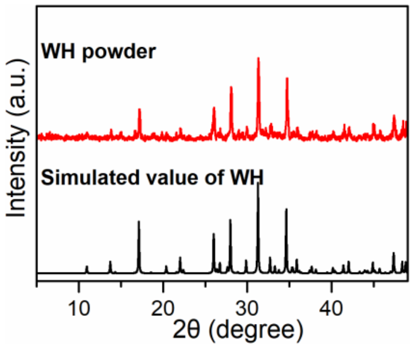

Figure S2. XRD patterns of WH powder and its simulation.

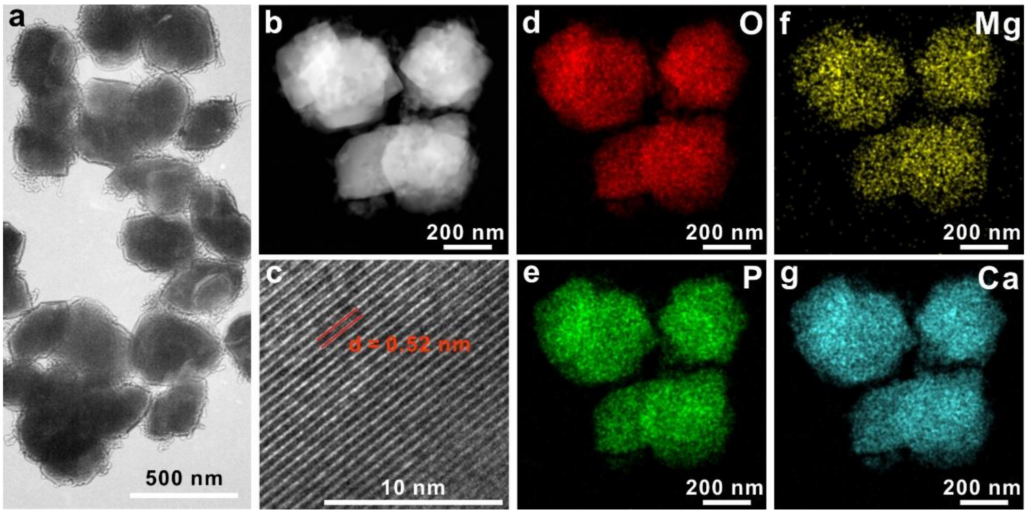

Figure S3. (a, b) Transmission electron microscopy (TEM) images of WH. (c) Lattice distance of WH confirmed by high-resolution TEM. (d-g) Energy-dispersive X-ray spectroscopy mapping of WH.

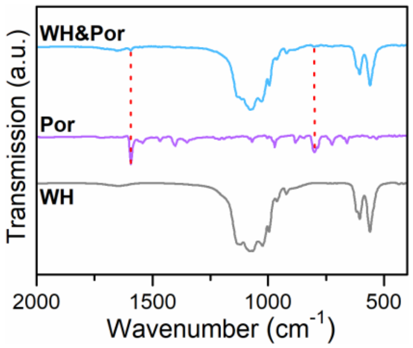

Figure S4. Fourier transform infrared (FT-IR) spectra of WH, Por and WH&Por.

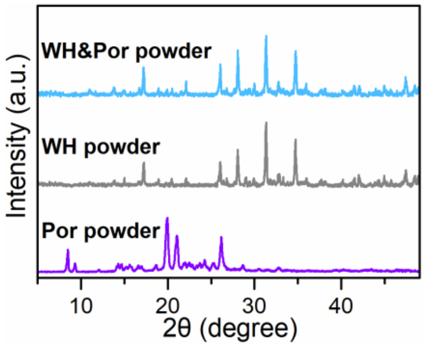

Figure S5. XRD patterns of Por, WH, and WH&Por.

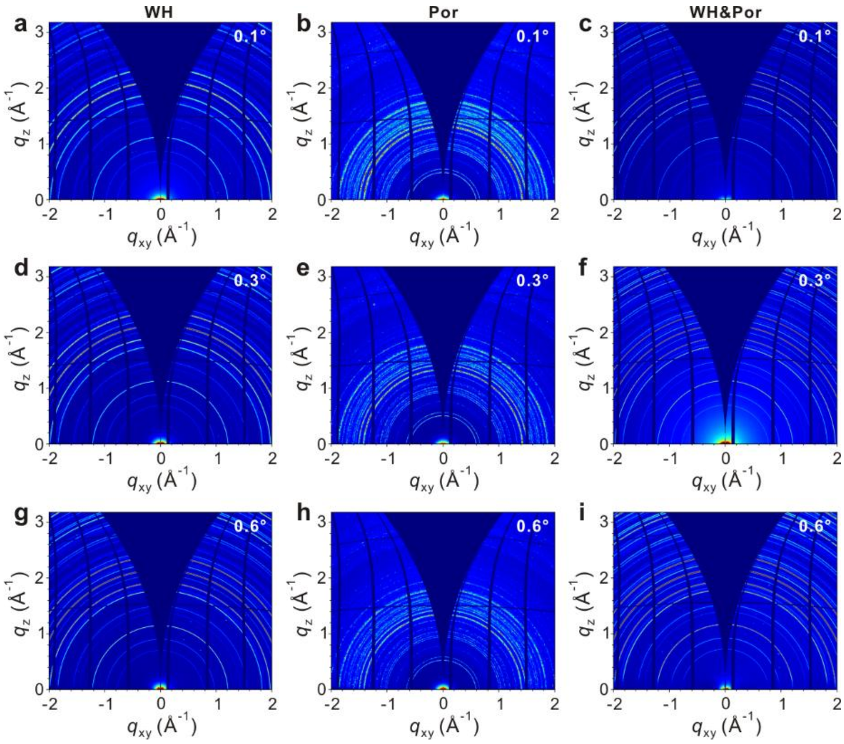

Figure S6. GIWAXS patterns of WH, Por and WH&Por samples at different grazing incidence angle.

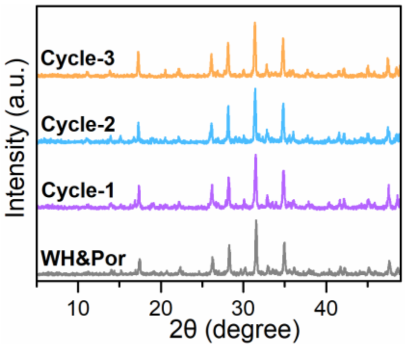

Figure S7. XRD patterns of WH&Por samples at different heating-cooling cycles.

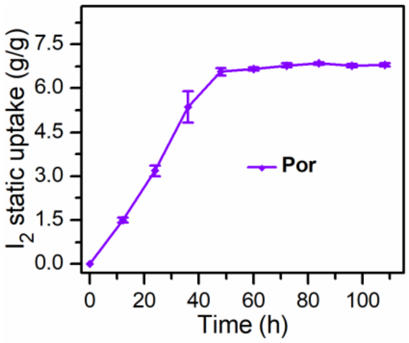

Figure S8. I_2 vapor uptake curve of Por at 348 K.

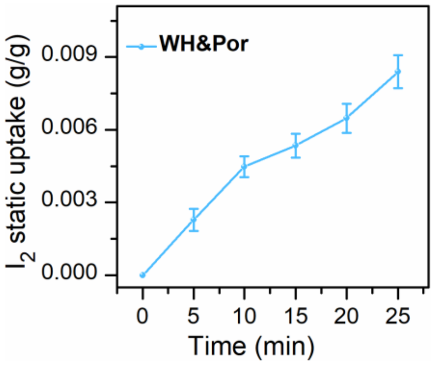

Figure S9. I_2 vapor uptake curve of WH&Por at 348 K.

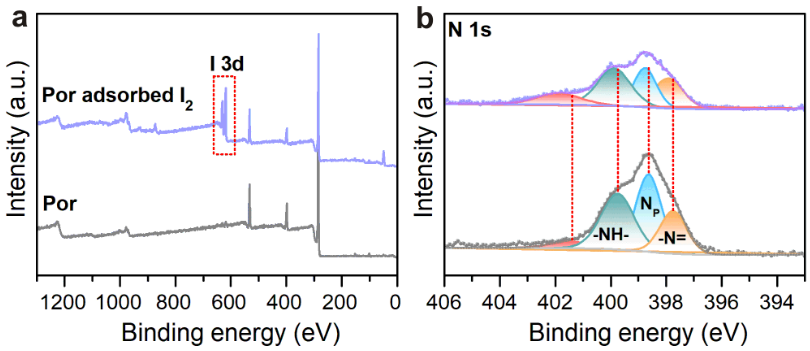

Figure S10. (a) Full survey X-ray photoelectron spectroscopy (XPS) spectra of Por and Por adsorbed I_2. (b) XPS spectra of N 1s of Por and Por adsorbed I_2. The -NH- and -N= refer to pyrrolic N of the porphyrin core in Por. The N_p refers to the pyridyl N.

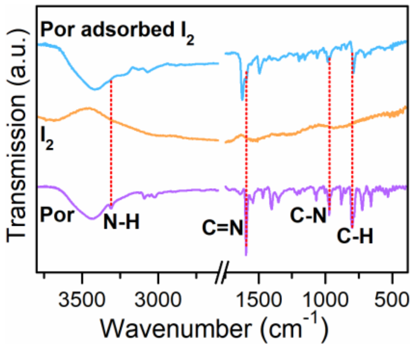

Figure S11. FT-IR spectra of Por, I_2 and Por adsorbed I_2.

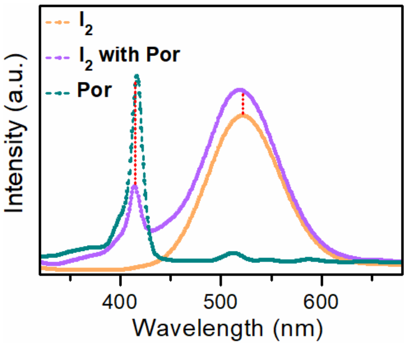

Figure S12. UV−vis absorption spectra of Por solution, I_2 solution and I_2 solution with Por solution.

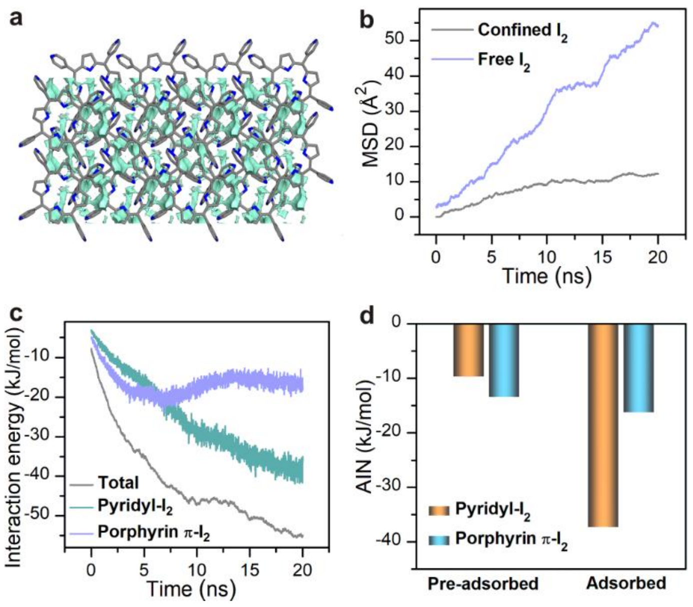

Figure S13. (a) Probability-density isosurface showing the most populated region of I_2 around the host porphyrin. (b) Comparison of the mean-square displacement (MSD) in free solution and inside the supramolecular cavity. (c) Time evolution of the interaction-energy components. (d) Averaged interaction-energy (AIN) contributions in the early (pre-adsorbed) and late (adsorbed) regimes.

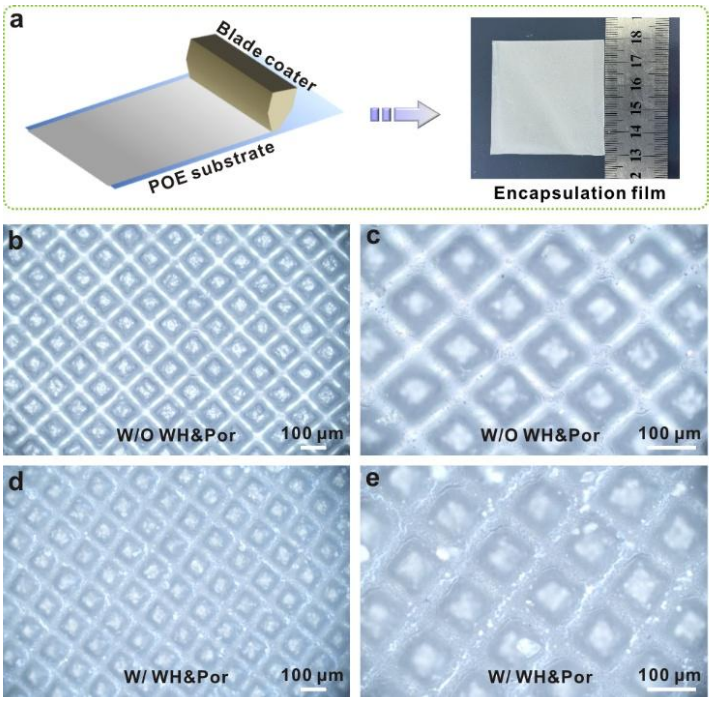

Figure S14. (a) Schematic illustration of encapsulation films preparation. Metallographic microscopy images of polyolefin elastomer (POE) films without (b,c) and with WH&Por (d,e).

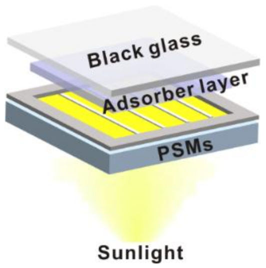

Figure S15. Schematic illustration of the encapsulated device structure.

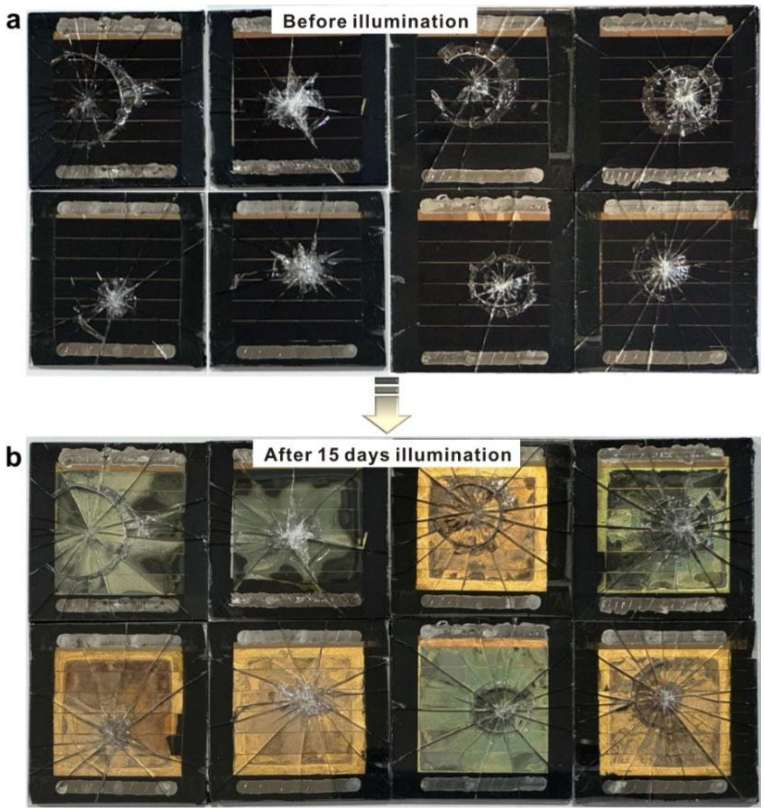

Figure S16. Photographs of the damaged 8-PSMs without WH&Por before and after illumination.

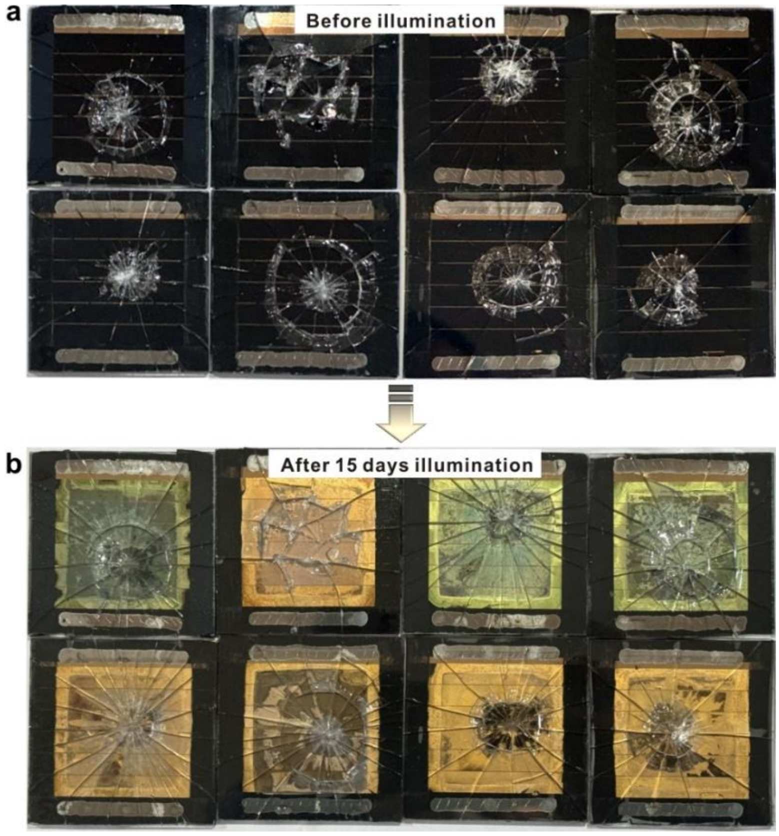

Figure S17. Photographs of the damaged 8-PSMs with WH&Por before and after illumination.

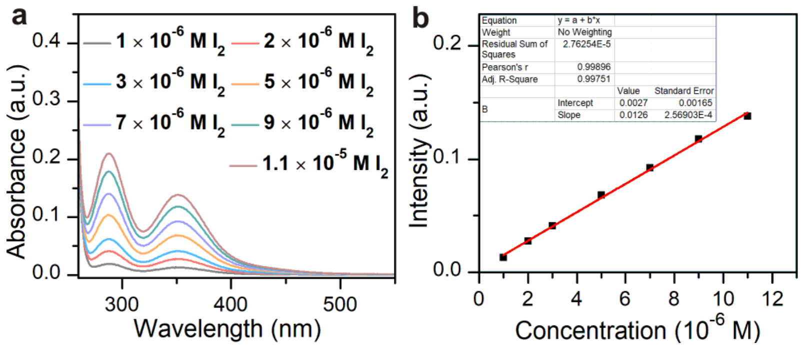

Figure S18. (a) UV-vis absorption spectra of triiodide ion at different concentration by adding iodine (I_2) into KI solution. (b) Relationship between the absorbance value at 351 nm and triiodide ion concentration.

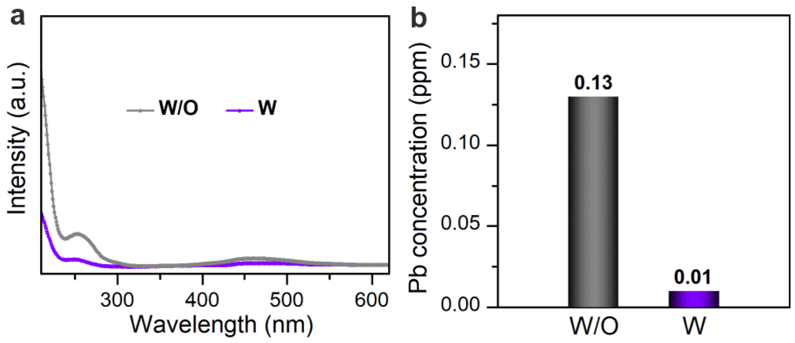

Figure S19. (a) UV-vis spectra of aqueous solutions collected from the test chambers (pH ≈ 4.5). (b) Quantitative analysis of Pb^{2+} concentrations in leachates under acidic aqueous conditions (pH ≈ 4.5).

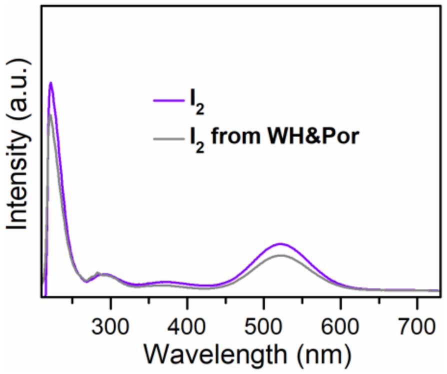

Figure S20. UV-vis absorption spectra of pristine I_2 and I_2 extracted from WH&Por.

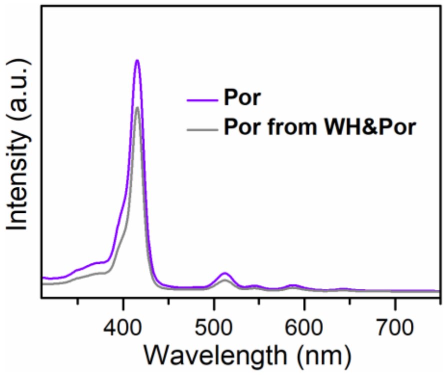

Figure S21. UV-vis absorption spectra of pristine Por and Por extracted from WH&Por.

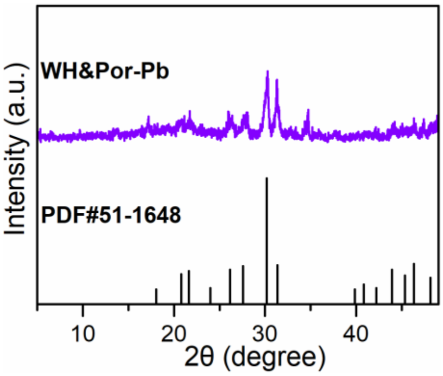

Figure S22. XRD patterns of hydroxypyromorphite (PDF#51-1648) and recovered WH&Por-based adsorbent (WH&Por-Pb).

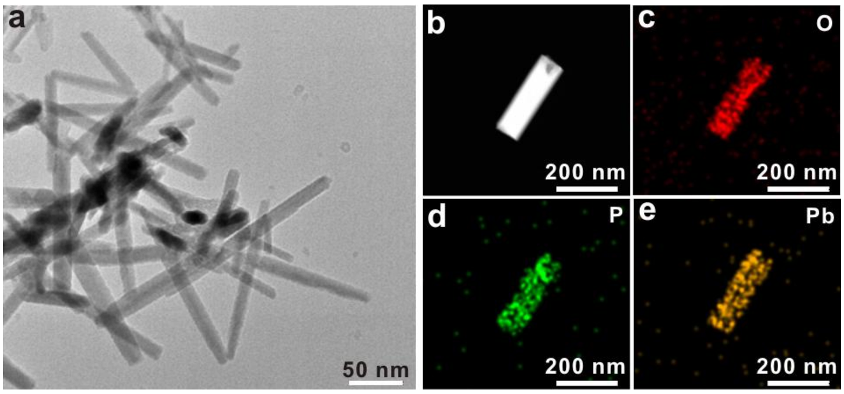

Figure S23. TEM images and energy-dispersive X-ray spectroscopy mapping of hydroxypyromorphite.

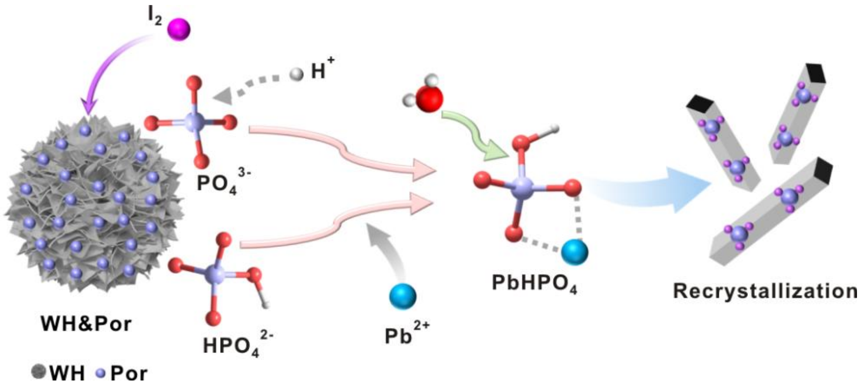

Figure S24. Schematic illustration of the dual-function adsorption mechanism of WH&Por for I_2 and Pb^{2+}.

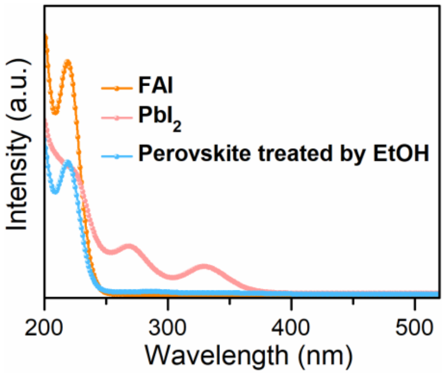

Figure S25. UV-vis absorption spectra of FAI, PbI_2 and perovskite films following ethanol (EtOH) immersion.

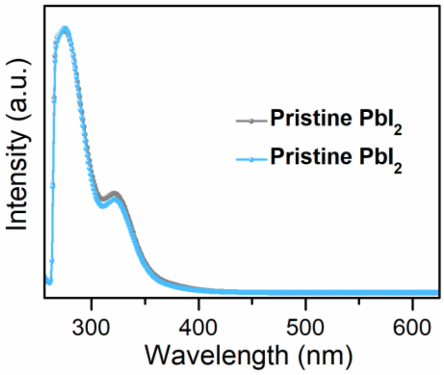

Figure S26. UV-vis absorption spectra of pristine and recycled PbI_2.

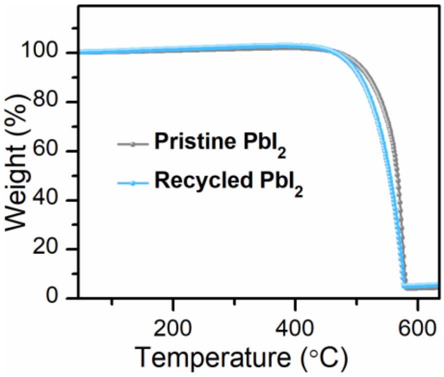

Figure S27. Thermal stability analysis of pristine and recycled PbI_2 via thermogravimetric analysis.

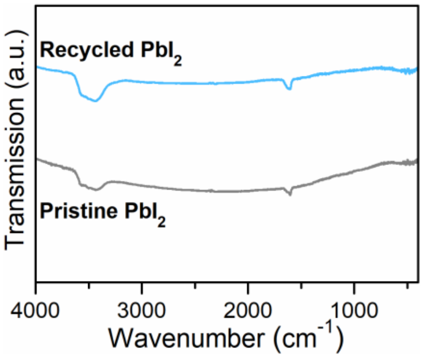

Figure S28. UV-vis absorption spectra of pristine and recycled PbI_2.

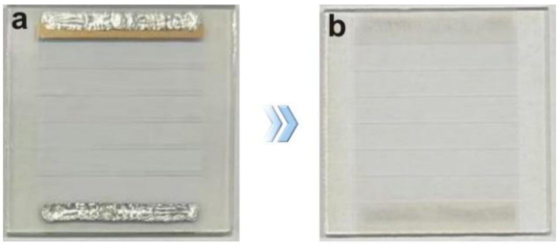

Figure S29. (a) Photograph of the FTO/SnO_2 substrate without cleaning. (b) Photograph of the recycled FTO/SnO_2 substrate.

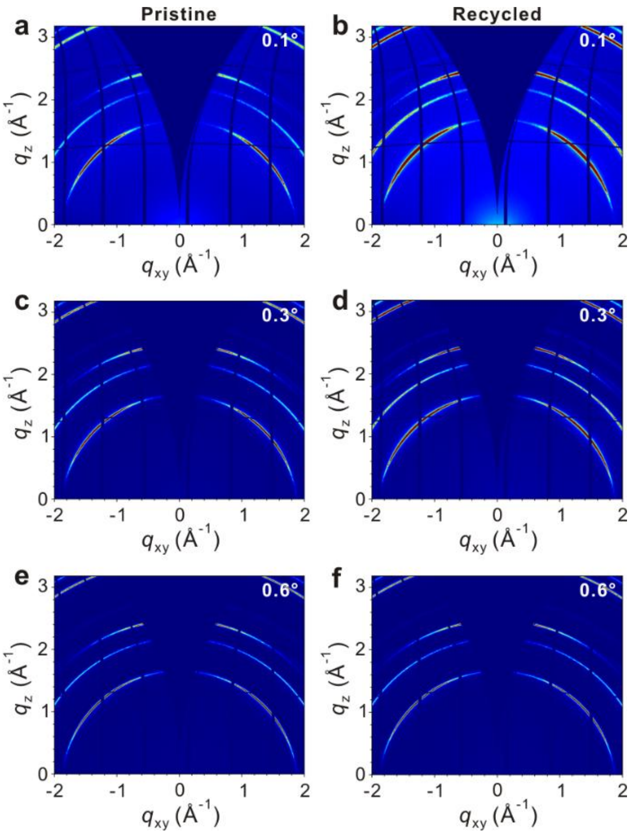

Figure S30. GIWAXS patterns of pristine and recycled FTO/SnO_2 substrate collected at different grazing incidence angle.

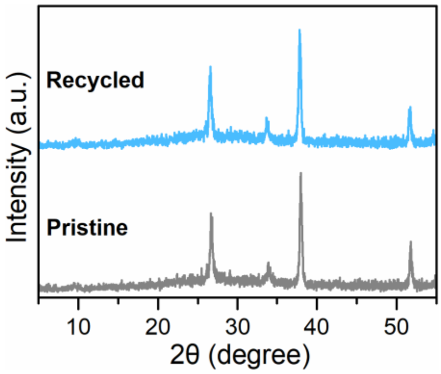

Figure S31. XRD patterns of pristine and recycled FTO/SnO_2 substrate.

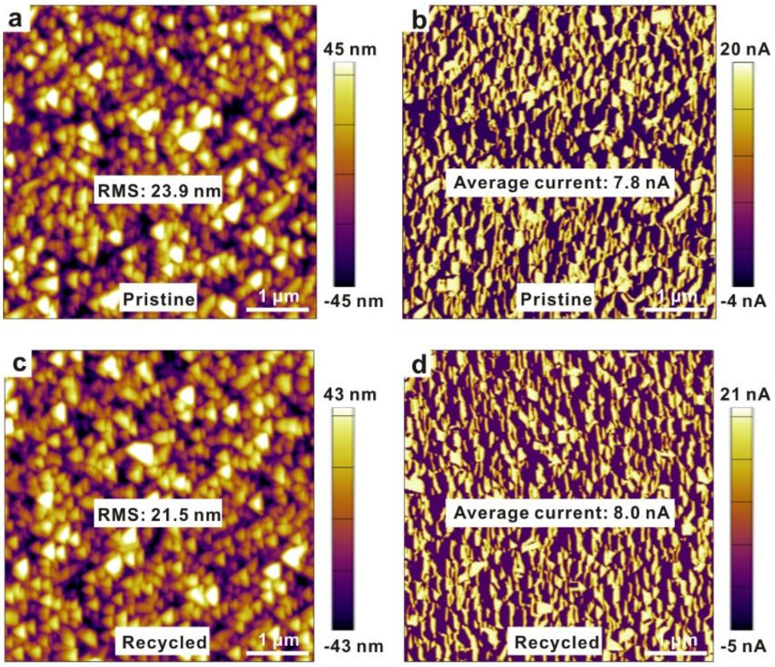

Figure S32. Conductive atomic force microscopy measurements of pristine and recycled FTO/SnO_2 substrate.

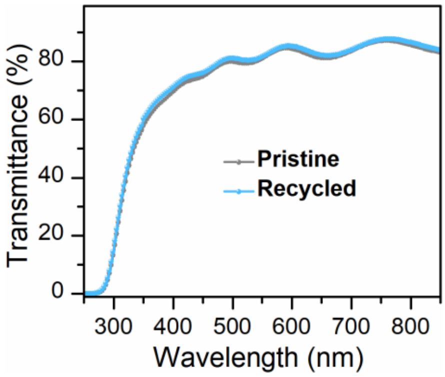

Figure S33. Transmission spectra of pristine and recycled FTO/SnO_2 substrate.

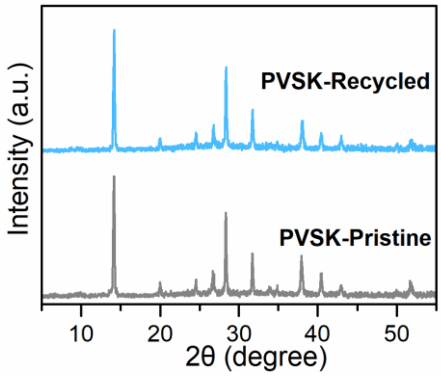

Figure S34. XRD patterns of perovskite (PVSK) films fabricated with pristine and recycled materials.

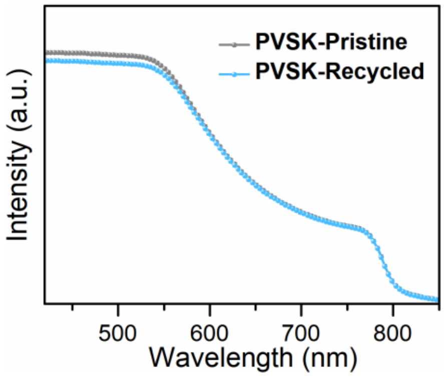

Figure S35. UV-vis absorption spectra of perovskite (PVSK) films prepared from pristine and recycled materials.

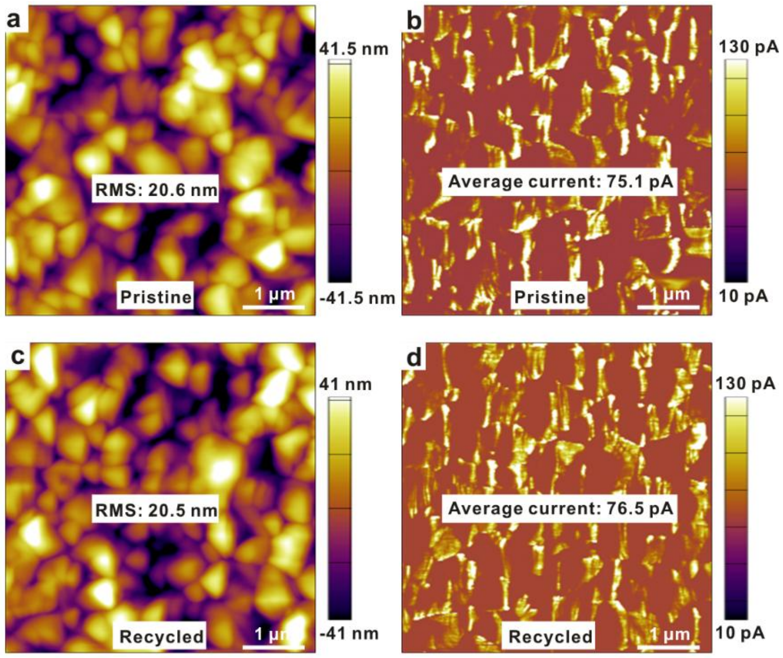

Figure S36. Conductive atomic force microscopy tests of perovskite films fabricated from pristine and recycled materials.

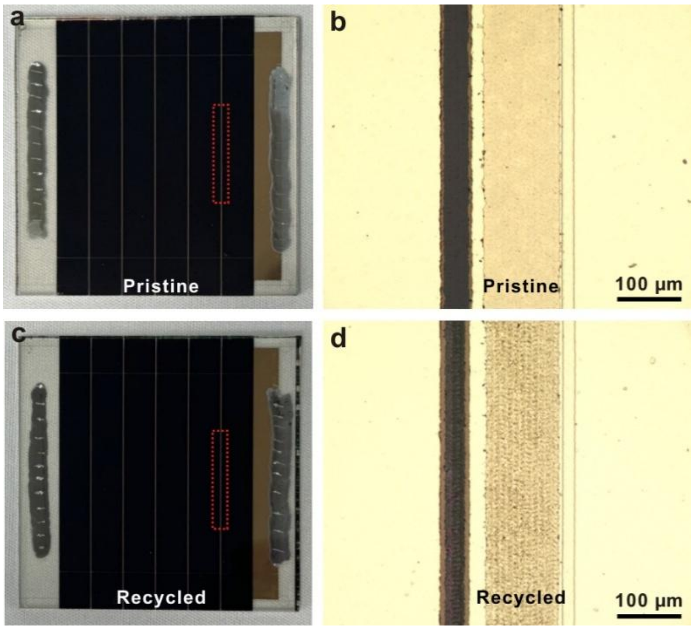

Figure S37. Photographs and micrographs of PSMs fabricated with pristine and recycled materials.

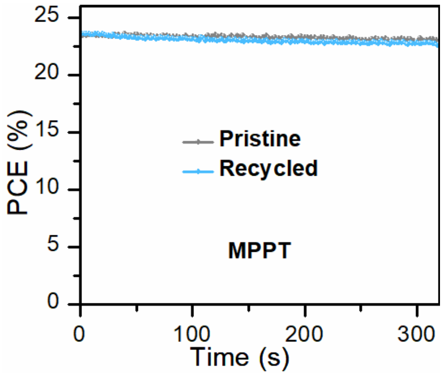

Figure S38. Maximum power point tracking performance of PSMs fabricated with pristine and recycled materials under AM 1.5 G illumination.

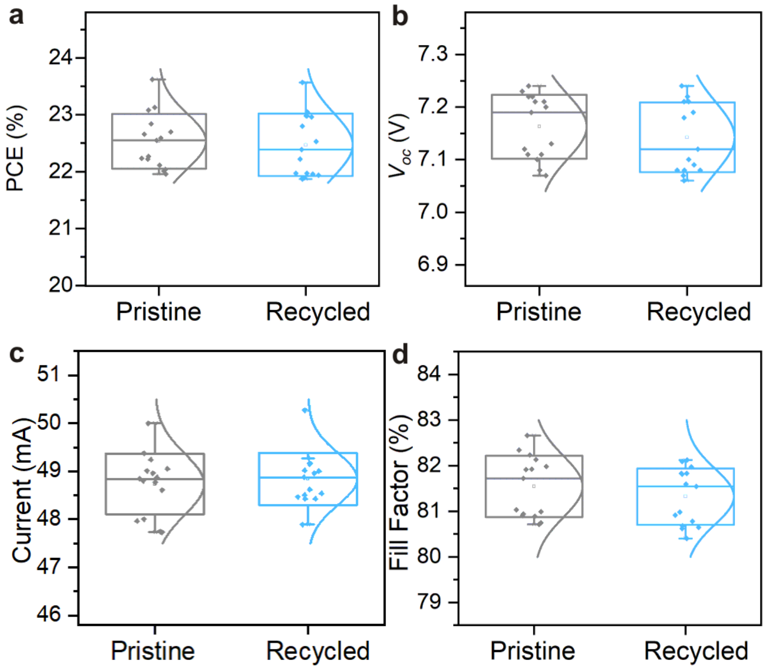

Figure S39. Power conversion efficiency (a), *Voc* (b), current (c), fill factor (d) of PSMs fabricated with pristine and recycled materials.

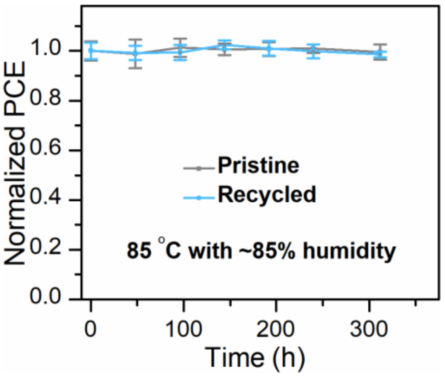

Figure S40. Stability of encapsulated devices fabricated from pristine and recycled materials. Devices were tested in a temperature- and humidity-controlled chamber at 85 °C and ~85% relative humidity. The initial power conversion efficiencies of devices based on pristine and recycled materials were 20.77 ± 0.80% and 20.62 ± 0.69%, respectively.

3. Supporting Tables

Table S1. Materials cost for PSMs (5 × 5 cm^2) based on our fabrication, with partial data appropriately adjusted according to relevant literature[11-13].

Component	Raw material	Price	Material cost ($)
FTO glass	FTO glass	112.36 $/m^2	0.281
Electron transport material	HCl	3.51 $/L	0.001
Electron transport material	$SnCl_2 \cdot 2H_2O$	314.96 $/kg	0.004
Electron transport material	Urea	315.62 $/kg	0.022
Electron transport material	Thioglycolic acid	526.62 $/L	0.001
Perovskite	PbI_2	280.90 $/kg	0.068
Perovskite	FAI	1123.60 $/kg	0.089
Perovskite	CsI	1032.30 $/kg	0.011
Perovskite	DMF	15.59 $/L	0.004
Perovskite	DMSO	27.95 $/L	0.002
Perovskite	EA	16.71 $/L	0.003
Hole transport materials	Spiro-OMeTAD	46.35 $/g	0.034
Hole transport materials	LiTFSI	0.46 $/g	0.043
Hole transport materials	Acetonitrile	25.14 $/L	0.001
Hole transport materials	Chlorobenzene	25.00 $/L	0.001
Hole transport materials	tBP	4.91 $/mL	0.001
Electrode	Au	139.68 $/g	0.279
Back glass	glass	3.51 $/m^2	0.009

The estimated materials cost for each PMS is about 0.854 USD per device.

Table S2. Materials cost and cumulative energy demand in the recycling process of PSMs, with partial data appropriately adjusted according to relevant literature and databases[11-15].

Component	Raw material	Price	Quantity of reagent	Cumulative energy demand (MJ)	Material cost ($)
Back glass	Ethanol	3.48 $/L	2 mL	0.06564	0.007
Hole transport materials and electrode	Dichloromethane	2.22 $/L	2 mL	0.0689	0.004
Por from WH&Por	Heptane	4.40 $/L	1 mL	0.03721	0.004
PbI_2 from WH&Por	HNO_3	6.18 $/L	0.1 mL	0.00195	0.0006
PbI_2 from WH&Por	KI	136.94 $/kg	2 mg	1.90E-05	0.0003
Organic components in perovskite	Ethanol	3.48 $/L	0.1 mL	0.00328	0.001
PbI_2	DMF	10.01 $/L	0.1 mL	0.00738	0.001
PbI_2	$NH_3 \cdot H_2O$	9.48 $/L	0.1 mL	6.51E-04	0.001
PbI_2	HI	196.34 $/L	0.1 mL	--	0.02
FTO/ETM	Ethanol	3.48 $/L	2 mL	0.06564	0.007

The estimated materials cost for each PMS is about 0.046 USD per device. Since no cumulative energy demand (CED) data for HI were found in the available literature and databases, the CED for HI was not included in the calculation.

Table S3. Energy cost in the recycling process of PSMs, with partial data appropriately adjusted according to relevant literature[11-13].

Process	Power (W)	Operating time (min)	Quantity of PSMs (piece)	Electricity (kW·h)	Cost ($)
Thermal delamination of encapsulated PSMs	1000	10	4	0.042	0.003
Recycle of HTM and electrode	1400	10	100	0.002	0.0002
Recycle of PbI_2	300	40	100	0.002	0.0002
Recycle of FTO/ETM	300	30	100	0.002	0.0002

The estimated energy cost for recycling each perovskite module is about 0.004 USD per device.

Table S4. Photovoltaic parameters of PSMs (aperture area of 12.63 cm^2) with pristine and recycled materials obtained in forward (FS) and reverse (RS) scans.

Devices		I_{sc}/mA	V_{oc}/V	FF/%	η/%
Pristine	RS	50.00	7.22	82.66	23.62
	FS	49.90	7.17	81.99	23.24
Recycled	RS	50.27	7.21	82.09	23.57
	FS	50.12	7.16	81.39	23.13

4. References

[1] H. L. Jang, K. Jin, J. Lee, Y. Kim, S. H. Nahm, K. S. Hong, K. T. Nam, *ACS nano* **2014**, *8*, 634-641.

[2] G. B. Xiao, X. Mu, Z. Y. Suo, X. Zhang, Z. Yu, J. Cao, *Angew. Chem. Int. Ed.* **2025**, *64*, e202414249.

[3] B. Chen, C. Fei, S. Chen, H. Gu, X. Xiao, J. Huang, *Nat. Commun.* **2021**, *12*, 5859-5868.

[4] S. Zhang, L. Shen, M. Huang, Y. Yu, L. Lei, J. Shao, Q. Zhao, Z. Wu, J. Wang, S. Yang, *ACS Sustainable Chem. Eng.* **2018**, *6*, 7558-7564.

[5] J. S. Hong, H. J. Kim, C. H. Sohn, O. Y. Gong, J. H. Choi, K. H. Cho, G. S. Han, K. T. Nam, H. S. Jung, *Energy Environ. Mater.* **2023**, *6*, e12374.

[6] C. Bannwarth, S. Ehlert, S. Grimme, *J. Chem. Theory Comput.* **2019**, *15*, 1652–1671.

[7] S. Grimme, C. Bannwarth, P. Shushkov, *J. Chem. Theory Comput.* **2017**, *13*, 1989-2009.

[8] J. Hutter, M. Iannuzzi, F. Schiffmann, J. VandeVondele, *WIREs Comput. Mol. Sci.* **2014**, *4*, 15-25.

[9] G. Bussi, D. Donadio, M. Parrinello, *J. Chem. Phys.* **2007**, *126*, 014101.

[10] E. Caldeweyher, J. M. Mewes, S. Ehlert, S. Grimme, *Phys. Chem. Chem. Phys.* **2020**, *22*, 8499-8512.

[11] Z. Li, Y. Zhao, X. Wang, Y. Sun, Z. Zhao, Y. Li, H. Zhou, Q. Chen, *Joule* **2018**, *2*, 1559-1572.

[12] L. Qiu, S. He, L. K. Ono, S. Liu, Y. Qi, *ACS Energy Lett.* **2019**, *4*, 2147-2167.

[13] Z. Wu, M. Sytnyk, J. Zhang, G. Babayeva, C. Kupfer, J. Hu, S. Arnold, J. Hauch, C. Brabec, I. M. Peters, *Energy Environ. Sci.* **2024**, *17*, 4248-4262.

[14] X. Tian, S. D. Stranks, F. You, *Nat. Sustain.* **2021**, *4*, 821-829.

[15] A. Amelio, G. Genduso, S. Vreysen, P. Luis, B. Van der Bruggen, *Green Chem.* **2014**, *16*, 3045-3063.
